# Single‐cell transcriptome analyses reveal disturbed decidual microenvironment in women of advanced maternal age

**DOI:** 10.1002/ctm2.70541

**Published:** 2025-12-17

**Authors:** Hongliang Xie, Yu Lu, Aolin Zhang, Anqi Zheng, Baofeng Rao, Cuiyu Yang, Anyao Li, Wenbo Guo, Linhua Hu, Xiaoling Huang, Chi Chiu Wang, Songying Zhang, Xiaohui Fan, Lu Li

**Affiliations:** ^1^ College of Pharmaceutical Sciences & Women's Hospital School of Medicine Zhejiang University Hangzhou China; ^2^ State Key Laboratory of Chinese Medicine Modernization College of Pharmaceutical Sciences Zhejiang University Hangzhou China; ^3^ Assisted Reproduction Unit Department of Obstetrics and Gynecology Sir Run Run Shaw Hospital Zhejiang University School of Medicine Hangzhou China; ^4^ Zhejiang Provincial Clinical Research Center for Reproductive Health and Disease Hangzhou China; ^5^ Zhejiang Key Laboratory of Precise Protection and Promotion of Fertility Hangzhou China; ^6^ Department of Nursing Sir Run Run Shaw Hospital Zhejiang University School of Medicine Hangzhou China; ^7^ Department of Obstetrics and Gynaecology Li Ka Shing Institute of Health Sciences School of Biomedical Sciences and Sichuan University‐Chinese University of Hong Kong Joint Reproductive Medicine Laboratory The Chinese University of Hong Kong Hong Kong China; ^8^ International Science and Technology Cooperation Base of Chinese Medicine Modernization and Big Health Innovation Center of Yangtze River Delta Zhejiang University Jiaxing China; ^9^ Modern Chinese Medicine and Reproductive Health Joint Innovation Center Innovation Center of Yangtze River Delta Zhejiang University Jiaxing China; ^10^ The Joint‐Laboratory of Clinical Multi‐Omics Research between Zhejiang University and Ningbo Municipal Hospital of TCM Ningbo Municipal Hospital of TCM Ningbo China

**Keywords:** advanced maternal age, decidualisation, decidua fibrosis, epithelial–mesenchymal transition, fibroblast–myofibroblast transition, PRR15‐TGF‐β axis, single cell RNA sequencing

## Abstract

**Background:**

Advanced maternal age (AMA) increases pregnancy risk. However, uterine‐specific mechanisms independent of oocyte and embryo quality remain poorly defined. This study aimed to characterise the decidual microenvironment in women with AMA to identify key pathological changes and regulatory pathways.

**Methods and results:**

Through integrated histology, organoid modelling, and high‐resolution scRNA‐seq of first‐trimester decidua from women of AMA and controlled reproductive age, we uncovered a pathologically remodelled decidual microenvironment characterised by aberrant cellular states and pathological differentiation pathways, leading to a pro‐fibrotic state and accompanied by immune cell dysfunction, and disrupted intercellular communication in the AMA decidua. Central to this pathology was hyperactivated TGF‐β signalling, driving fibroblast‐to‐myofibroblast transition and extracellular matrix overproduction, thereby fuelling fibrosis. Aberrant TGF‐β further impaired decidual stromal cell (DSC) differentiation, leading to the failure of the essential mesenchymal‐to‐epithelial transition. We identified PRR15 as a novel DSC‐specific regulator that is markedly suppressed in AMA. PRR15 deficiency unleashed hyperactive TGF‐β/SMAD signalling, directly causing decidualisation failure, enhanced fibrosis, and aborted DSC differentiation. Epithelial–mesenchymal transition and immune cell reprogramming towards pro‐fibrotic transcriptional signatures further amplify the fibrotic pathology.

**Conclusion:**

This study established the aged decidual microenvironment, orchestrated by dysregulated TGF‐β signalling and PRR15 loss, as a critical independent determinant of reproductive failure in AMA. Thus, it unveils novel diagnostic and therapeutic targets and strategies.

**Key points:**

- We provide the first single‐cell atlas of the human decidua in advanced maternal age (AMA).
- A novel PRR15‐TGF‐β axis is identified, where PRR15 loss drives stromal fibrosis and decidualisation failure.
- This study reveals that AMA‐associated uterine fibrosis begins in the first trimester, shifting focus to maternal factors.

## Graphical abstract

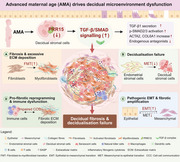

## 1 INTRODUCTION

Advanced maternal age (AMA) is an escalating global health challenge that profoundly impacts reproductive outcomes.^1^ Declining oocyte quality, particularly aneuploidy, has long been considered the primary driver of adverse pregnancy outcomes in older women.^2^ However, the persistently high rates of pregnancy complications, including gestational hypertension, pre‐eclampsia, post‐partum haemorrhage and twin pregnancies, persist despite advancements in assisted reproductive technologies.^3^, ^4^, ^5^ This suggests that the oocyte‐centric view is incomplete, consequently highlighting the need to investigate other maternal factors that contribute to age‐related reproductive decline.

In addition to the oocyte, the maternal uterine environment is indispensable for successful embryo implantation, placentation and pregnancy maintenance.^6^, ^7^ The decidua, a specialised uterine lining, provides a vital interface for embryo‐maternal crosstalk and a supportive microenvironment.^8^, ^9^ Its proper formation and function are prerequisites for a healthy pregnancy, increasing its role from that of a passive recipient to an active and critical determinant of reproductive success.^10^, ^11^ This underscores the importance of understanding how the ageing uterine environment, particularly the decidua, contributes to the unique challenges faced by women of AMA. Therefore, our study aimed to characterise the decidual microenvironment in women of AMA to identify key pathological changes and regulatory pathways.

To address this critical knowledge gap, we generated an unprecedented, high‐resolution single‐cell transcriptomic atlas of the first‐trimester decidua from women of AMA and compared it with that of the control reproductive age (CTR). While single‐cell RNA sequencing (scRNA‐seq) has recently illuminated the general aspects of decidual biology in recurrent miscarriage, obstetric antiphospholipid syndrome and preeclampsia, no comprehensive analysis has specifically elucidated the multi‐faceted pathological changes within the decidual microenvironment in women with AMA.^12^, ^13^, ^14^, ^15^, ^16^, ^17^

Our scRNA‐seq analysis revealed a pathologically remodelled decidual microenvironment in women of AMA, characterised by aberrant fibroblast expansion, immune cell depletion and disrupted intercellular communication networks. Importantly, we discovered a novel stromal‐cell‐specific regulator whose significant downregulation in AMA is mechanistically linked to TGF‐β signalling dysregulation and subsequent functional impairment. This integrated understanding enhances our understanding of age‐related reproductive decline. Additionally, it highlights the decidual microenvironment, along with oocyte quality, as a critical independent determinant of reproductive success in women of AMA. Our findings open new avenues for diagnostics and multi‐target therapeutic strategies for AMA‐related complications.

## 2 MATERIALS AND METHODS

### 2.1 Patient recruitment and sample collection

This study received ethical approval from the Ethics Committee of Sir Run Run Shaw Hospital, Zhejiang University School of Medicine (approval no.: 2024 Research Ethics Review No. 0761). All participants provided written informed consent. A total of 18 decidual samples were obtained from women undergoing elective first‐trimester surgical termination. These samples were divided into two distinct cohorts: (1) Discovery Cohort (scRNA‐seq): Samples from three women of AMA (35–49 years) and three women of CTR (22–30 years) were used for single‐cell transcriptomic profiling. Clinical characteristics for this cohort are detailed in Table S1. (2) Validation Cohort: Samples from an additional six women of AMA and six women of CTR were collected for histological and functional validation experiments. Clinical characteristics for this cohort are detailed in Table S2. Owing to variations in sample quality and the technical requirements of different assays, the number of biological replicates from the validation cohort used for each experiment varied. The precise number of replicates for each analysis is explicitly stated in the corresponding figure legend. Exclusion criteria included recent hormonal therapy, genetic disorders, reproductive pathologies, or chronic diseases; patients aged 31–34 years were excluded to preclude age spectrum overlap.

### 2.2 Cell and organoid culture

#### 2.2.1 Isolation and culture of primary stromal cells and endometrial epithelial organoids (EEOs)

EEOs and stromal cells were established as previously described.^18^ Briefly, decidual tissue fragments were digested in RPMI 1640 medium (Gibco; 11875093) containing 10% FBS (Gibco; 10099141),.4 mg/mL collagenase IV (Sigma; C‐9263) and 1.25 U/mL dispase II (Sigma; D4693). Epithelial fragments retained on a 100 µm strainer (Falcon; 352360) were resuspended in ice‐cold Matrigel (Corning; 356231) at a 1:20 ratio, plated as 20 µL droplets in 48‐well plates (Costar; 3548) and cultured in organoid Expansion Medium (ExM). ExM composition is provided in Table S3. Stromal cells were collected from flow‐through, treated with RBC lysis buffer (Beyotime; C3702) and cultured in DMEM/F12 (Gibco; 11330032) + 10% FBS + 1% penicillin/streptomycin (Gibco; 15140122).

#### 2.2.2 Hormonal stimulation of EEOs and stromal cells

Hormonal stimulation protocols for EEOs and stromal cells were based on Burton's and Borodkina's team.^18^, ^19^ Briefly, EEOs were primed with 10 nM β‐oestradiol (E2; Sigma; E4389) for 48 h, then stimulated with 10 nM E2, 1 µM progesterone (P4; Sigma; P7556), and 1 µM 8‐bromoadenosine 3′,5′‐cyclic monophosphate (cAMP; Sigma; B7880) for 96 h to mimic secretory‐phase conditions. Stromal cells were decidualised using 0.5 mM cAMP and 1 µM medroxyprogesterone acetate (MPA; MedChemExpress, HY‐B0469R) in phenol red‐free DMEM/F12 medium (Gibco; 21041025) with 2% charcoal‐stripped FBS (OPCEL; BS‐1201) for 4 days.

### 2.3 Histopathology and immunostaining

#### 2.3.1 Histopathology

Human decidual tissues were fixed in 4% paraformaldehyde (PFA; Beyotime; P0099) for 24 h, embedded in paraffin, and sectioned into 4 µm. Sections were stained with hematoxylin and eosin (H&E), Masson's trichrome, and picric acid‐saturated Sirius Red. All slides were examined using an Olympus BX61 light microscope.

#### 2.3.2 Immunofluorescence (IF) staining

For IF, sections (5 µm) underwent antigen retrieval in citrate buffer (pH 6.0) at 95°C for 15 min. Cells were fixed with 4% PFA, permeabilised with 0.3% Triton X‐100 (Sigma; T8787), and blocked with 5% bovine serum albumin (Gibco; 15260037). Primary antibodies (Table S4) were incubated overnight at 4°C, followed by Alexa Fluor 488‐conjugated goat anti‐rabbit immunoglobulin (IgG) (Beyotime; A0423), Alexa Fluor 647‐conjugated goat anti‐mouse IgG (Beyotime; A0460) and Alexa Fluor 647‐conjugated goat anti‐rabbit IgG (Beyotime; A0468). Nuclei were counterstained with DAPI (Beyotime; C1002). Images were acquired using an Olympus BX61 and Leica TCS SP8 confocal microscope.

#### 2.3.3 Immunohistochemistry (IHC) staining

IHC was performed on deparaffinised sections (5 µm) with antigen retrieval via microwaving in sodium citrate buffer (pH 6.0). Endogenous peroxidase was quenched with 3% H_2_O_2_ at room temperature. Primary antibodies (Table S4) were incubated overnight, followed by HRP‐conjugated secondary antibody (Beyotime; A0208) and DAB (Beyotime; P0202) visualisation. Imaging was performed using an Olympus BX61 microscope.

### 2.4 Gene manipulation and mechanistic studies

#### 2.4.1 Lentiviral‐mediated proline rich 15 (PRR15) knockdown (KD‐PRR15)

Stromal cells were transduced with lentiviral particles (Beijing Qingke Biotechnology Co., Ltd.) carrying shRNA targeting PRR15 (KD‐PRR15; 5′‐TGGAAATCGCTCACCAACA‐3′) or a non‐targeting control (KD‐NC; 5′‐ACTACCGTTGTTATAGGTGT‐3′). Transductions were performed at a multiplicity of infection of 50 in complete medium supplemented with 8 µg/mL polybrene. After 48 h, stably transduced cells were selected with.5 µg/mL puromycin for five days. Knockdown efficiency was validated using RT‐qPCR.

#### 2.4.2 PRR15 overexpression (OE‐PRR15)

The full‐length coding sequence of human PRR15 (Table S5) was cloned into the pLV‐ZsGreen(2A) PURO‐CMV vector (Beijing Qingke Biotechnology Co., Ltd.). The resulting construct (OE‐PRR15) and an empty vector control (OE‐NC) were verified using Sanger sequencing and then transfected into stromal cells using Lipofectamine 3000. Overexpression was confirmed 48 h post‐transfection using RT‐qPCR.

#### 2.4.3 TGF‐β pathway inhibition and rescue assay

To assess the functional role of TGF‐β signalling, primary stromal cells from the AMA validation cohort were treated with 10 µM SB431542 (MedChemExpress; HY‐10432), a specific inhibitor of the TGF‐β type I receptor, or vehicle (0.1% DMSO) for 48 h before analysis. Cells were then harvested for RNA extraction and subsequent RT‐qPCR analysis. For the mechanistic rescue experiment, stable PRR15‐knockdown cells were decidualised for 96 h in the presence or absence of 10 µM SB431542. The rescue of decidualisation and pathway inhibition was assessed by enzyme‐linked immunosorbent assay (ELISA) for secreted insulin‐like growth factor binding protein‐1 (IGFBP1) and IF for nuclear SMAD2/3.

### 2.5 Molecular biology assays

#### 2.5.1 Western blot analysis

Proteins from stromal cells were extracted using radioimmunoprecipitation assay buffer (Beyotime; P0013B) supplemented with a cocktail of protease and phosphatase inhibitors (Beyotime; P1045). Samples (30 µg/lane) were separated using sodium dodecyl sulfate–polyacrylamide gel electrophoresis, transferred to polyvinylidene fluoride (Millipore; IPVH00010) membranes, and probed with antibodies against IGFBP1, phosphorylated SMAD family member 2 (p‐SMAD2), phosphorylated SMAD family member 3 (p‐SMAD3), SMAD family member 2 (SMAD2), SMAD family member 3 (SMAD3), Vimentin (VIM), collagen type VI alpha 1 chain (COL6A1), glyceraldehyde‐3‐phosphate dehydrogenase (GAPDH) and alpha‐tubulin (ɑ‐tubulin) (Table S4). HRP‐conjugated secondary antibodies (Beyotime; A0216; 1:5000) were detected using chemiluminescence. Band intensities were quantified using ImageJ (NIH, Version 1.53k).

#### 2.5.2 Real‐time quantitative PCR (RT‐qPCR) analysis

Total RNA was isolated using the RNeasy Mini Kit (Qiagen; 74104). cDNA was synthesised from 1 µg of RNA, and RT‐qPCR was performed using SYBR Green Master Mix (Yeasen Biotech; 11184ES08) with gene‐specific primers (Sangon Biotech; Table S6). Reactions were run in triplicate on a CFX96 Touch system (Bio‐Rad Laboratories). Relative mRNA levels of *IGFBP1*, prolactin (*PRL*), *PRR15*, actin alpha 2 (*ACTA2*) and collagen type 1 alpha1 chain (*COL1A1*) were normalised to β‐actin via the 2^−ΔΔCt^ method.

#### 2.5.3 ELISA analysis

Concentrations of secreted active TGF‐β1 and IGFBP1 in cell culture supernatants were quantified using commercial human ELISA kits (TGF‐β1, cloud‐clone, SEA124Hu; IGFBP1, cloud‐clone, SEA052Hu) according to the manufacturer's protocol.

### 2.6 Single‐cell RNA sequencing and data analysis

#### 2.6.1 Cell dissociation and library preparation

Human decidual tissues were enzymatically dissociated using a Human Tumor Dissociation Kit (Miltenyi Biotec; 130‐095‐929) on a gentleMACS Octo Dissociator (Miltenyi Biotec; 130‐096‐427). Cell suspensions were filtered through 70 µm strainers (Miltenyi Biotec; 130‐101‐262), treated with RBC lysis buffer (Miltenyi Biotec; 130‐094‐183), and dead cells were removed. Viability was confirmed >85% by trypan blue exclusion. Libraries were prepared using the 10x Genomics Chromium Next GEM Single Cell 3′Kit v3.1 (10x Genomics; 1000121) and sequenced on an Illumina NovaSeq 6000 (150‐bp paired‐end reads).

#### 2.6.2 Data pre‐processing, quality control and integration

Raw sequencing data were processed using CellRanger (v7.1.0, 10x Genomics) to generate feature‐barcode matrices against the human reference genome (GRCh38). The resultant matrices were loaded into R (v4.5.1) and processed using the Seurat package (v5.3.0).^20^ Low‐quality cells were filtered, retaining those with a feature count (nFeature_RNA) between 1000 and 10 000, and less than 10% mitochondrial gene content. Doublets were identified and removed using the DoubletFinder package (v2.0.6).^21^ A summary of the cell numbers and quality metrics for each sample after initial processing is provided in Table S7. After quality control filtering and doublet removal for each sample, the individual datasets were normalised using Seurat's NormalizeData function and then merged. To correct for batch effects between patients, the merged dataset was integrated using the Harmony algorithm (v1.2.3) implemented in Seurat.^22^

#### 2.6.3 Dimensionality reduction, clustering and annotation

Principal component analysis was performed on the integrated data, and the top 50 principal components (PCs) were selected for downstream analyses. Cell clusters were identified using the FindClusters function with a resolution parameter of.8. For visualisation, uniform manifold approximation and projection (UMAP) was performed on the top 50 PCs. For high‐resolution subcluster analysis, the fibroblast (FB), decidual stromal cell (DSC), and endometrial epithelial cell (EEC) populations were subsetted from the integrated object. Clustering was then repeated on each subset using the Louvain algorithm with resolution parameters of.1 (for FB),.2 (for DSC), and.2 (for EEC). To ensure the robustness of our subclusters, the Leiden algorithm^23^ was also employed at various resolutions for comparison. Cell populations were manually annotated based on the expression of established canonical marker genes.

#### 2.6.4 Differential expression gene and functional enrichment analysis

Differential expression gene (DEG) analysis between AMA and CTR groups within each cell type was performed using the Wilcoxon rank‐sum test (FindMarkers function in Seurat) with a significance threshold of adjusted *p*‐value < .05, absolute log_2_ (fold change) >.25, and expression detected in at least 25% of cells within the clusters. This log_2_FC threshold is a well‐established standard for single‐cell analysis to capture biologically meaningful shifts while minimising noise.^24^, ^25^, ^26^ Detailed statistics for key DEGs discussed in the manuscript are provided in Table S8. Gene Ontology (GO) and Kyoto Encyclopedia of Genes and Genomes (KEGG) pathway enrichment analyses were conducted using the clusterProfiler package (v4.16.0).^27^ Gene signature scores for processes like ‘TGF‐β Signalling’ and ‘EMT’ were calculated using the AddModuleScore function in Seurat, with gene sets sourced from MSigDB Hallmark collections.^28^

#### 2.6.5 Trajectory and cell–cell communication analysis

Cellular differentiation trajectories for FB, DSC, and EEC subsets were inferred using the R package Slingshot (v2.8.0),^29^ run on the previously computed UMAP embeddings for each cell population. Intercellular communication networks were analysed using CellChat (v2.2.0)^30^ with the CellChatDB.human database. Networks for CTR and AMA groups were compared to identify dysregulated signalling pathways.

### 2.7 Statistical analysis

All statistical analyses for in vitro and histological data were performed using GraphPad Prism 9.0 (GraphPad Software, La Jolla, CA). Data are presented as mean ± standard deviation (SD) or mean ± standard error of the mean (SEM) as explicitly indicated in the corresponding figure legends. Comparisons between two groups were made using an unpaired, two‐tailed Student's *t*‐test. Comparisons among multiple groups were performed using one‐way analysis of variance (ANOVA) with Tukey's post hoc test. Statistical methods for scRNA‐seq analyses (e.g., Wilcoxon rank‐sum test for DEG analysis and cell proportion comparisons) are detailed in the sections above and in the figure legends. A *p*‐value < .05 was considered statistically significant.

## 3 RESULTS

### 3.1 AMA decidua exhibits impaired stromal differentiation and fibrotic remodelling

**FIGURE 1:**
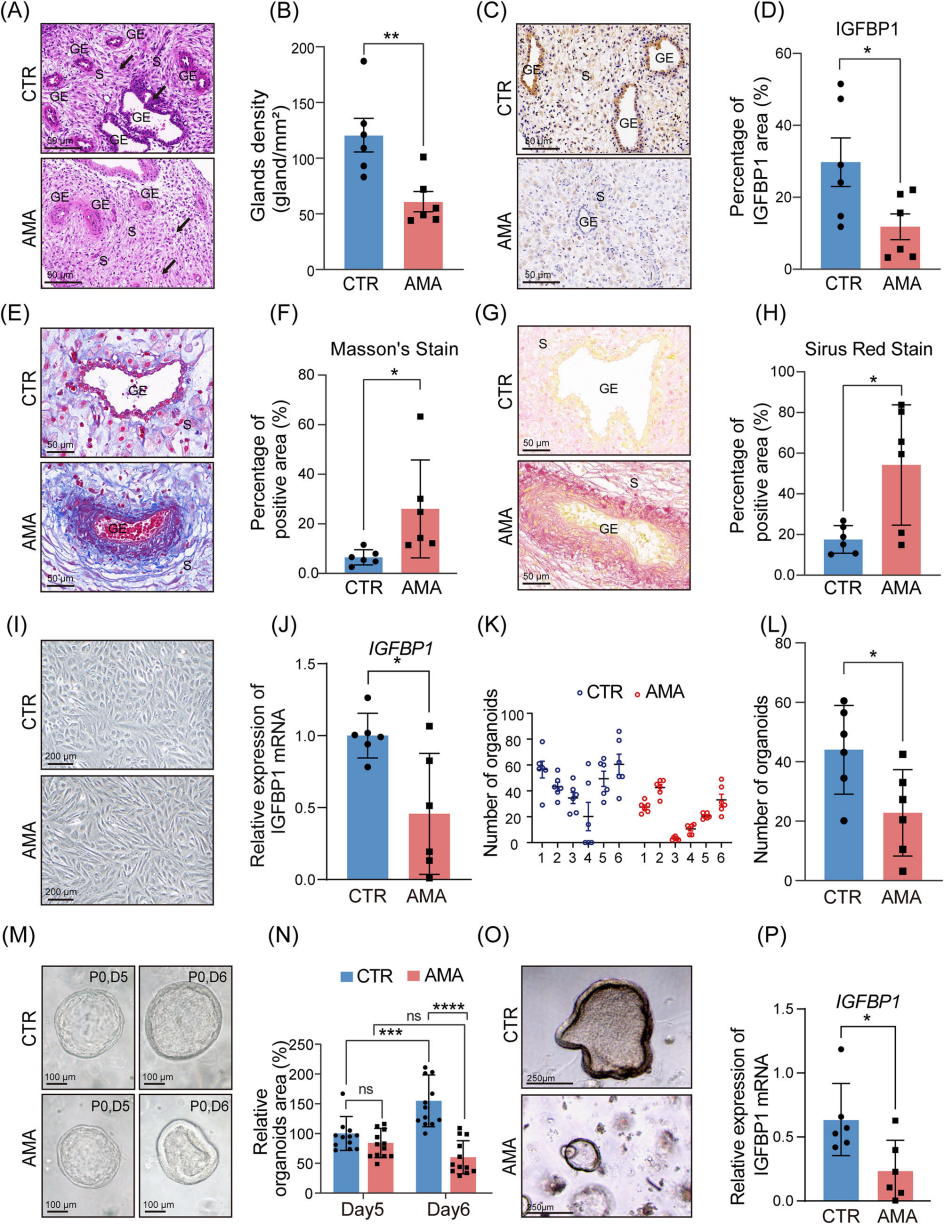
Women of AMA associate with structural and functional decidual deficits. (A) Representative H&E‐stained decidual sections from CTR (top panel) and AMA (bottom panel) groups. The arrow indicates a region in the AMA stroma with nuclear pleomorphism and architectural disorganisation. S, stroma; GE, glandular epithelium. Scale bar: 100 µm. (B) Quantification of gland density (glands/mm^2^) from H&E‐stained sections (*n* = 6 biological replicates per group). (C) Representative IHC for the decidualisation marker IGFBP1. Scale bar: 100 µm. (D) Quantification of IGFBP1 staining intensity from IHC (*n* = 6 biological replicates per group). (E) Representative Masson's trichrome staining showing collagen deposition (blue). Scale bar: 50 µm. (F) Quantification of collagen‐positive area from (E) (*n* = 6 biological replicates per group). (G) Representative Sirius Red staining showing collagen deposition (red). Scale bar: 50 µm. (H) Quantification of collagen‐positive area from (G) (*n* = 6 biological replicates per group). (I) Representative morphology of stromal cells isolated from CTR and AMA decidua after 4 days in vitro decidualisation with 1 µM MPA and 0.5 mM cAMP. Scale bar: 200 µm. (J) RT‐qPCR analysis of *IGFBP1* mRNA in decidualised stromal cells (*n* = 6 biological replicates per group). (K) Detailed plot showing EEO formation results from individual patients. Each point represents a technical replicate, grouped by biological replicate (*n* = 6 biological replicates per group). (L) Summary quantification of EEO formation efficiency (*n* = 6 biological replicates per group). (M) Representative images of EEO growth between day 5 and day 6 of passage 0. Scale bar: 100 µm. (N) Quantification of the change in relative organoid area, calculated as the ratio of an organoid's area on day 6 to its area on day 5 (area day 6/area day 5 × 100%, *n* = 3 biological replicates per group, with four organoids measured per patient). (O) Representative morphology of hormone‐stimulated EEOs from CTR (top panel) and AMA (bottom panel) groups. Scale bar: 250 µm. (P) RT‐qPCR analysis of *IGFBP1* expression in hormone‐stimulated EEOs (*n* = 6 biological replicates per group). Data in bar graphs are presented as the mean ± SD. Statistical significance was determined by a two‐tailed unpaired Student's *t*‐test (*p < 0.05, **p < 0.01, ****p < 0.0001).

To characterise the decidua in women of AMA, we first performed comprehensive histological and functional analyses. H&E staining of AMA decidua showed impaired glandular architecture, evidenced by disorganised luminal structures (Figure 1A, GE) and a significantly reduced endometrial gland density compared with CTR samples (Figure 1B). Furthermore, decidual stromal cells in the AMA group displayed notable nuclear pleomorphism and architectural disorganisation (Figure S1A, arrow), suggesting defective differentiation. Consistent with these morphological changes, IHC confirmed a marked downregulation of the decidualisation marker IGFBP1 in AMA tissues (Figure 1C,D). (Figure 1C,D). A key pathological feature identified was extensive tissue fibrosis. Both Masson's trichrome and Sirius Red staining revealed excessive and disorganised collagen deposition throughout the stroma in the AMA group (Figure 1E,G), a finding confirmed by quantitative analysis of the stained areas (Figure 1F,H).

We further assessed whether these structural defects translated to functional impairments using in vitro models. Primary stromal cells isolated from individuals of AMA exhibited impaired decidualisation, retaining a fibroblastic morphology (Figure 1I) and showing a blunted induction of key decidualisation marker genes *IGFBP1* and *PRL* upon hormonal stimulation (Figure 1J; Figure S1B). The epithelial compartment was also functionally compromised. EEOs (Figure S1C) derived from AMA tissue demonstrated significantly reduced primary formation efficiency (Figure 1K,L; Figure S1D); representative images show AMA‐derived EEOs grew more slowly (Figure 1M), and quantitative analysis confirmed a significantly lower relative organoid area (Figure 1N) and a reduced growth rate compared with CTR EEOs (Figure S1E). Finally, the response of EEOs to hormonal stimulation was impaired in the AMA group; unlike the thickened, secretory‐like columnar epithelium formed in CTR EEOs (Figure 1O, top panel), AMA EEOs failed to undergo this morphological transition (Figure 1O, bottom panel) and showed significantly reduced *IGFBP1* expression (Figure 1P). Collectively, these findings establish that the AMA decidua represents a profoundly dysfunctional microenvironment characterised by extensive fibrosis and both stromal and epithelial impairment.

### 3.2 Single‐cell atlas and validation analyses reveal a pro‐fibrotic, immune‐depleted state in AMA decidua

**FIGURE 2:**
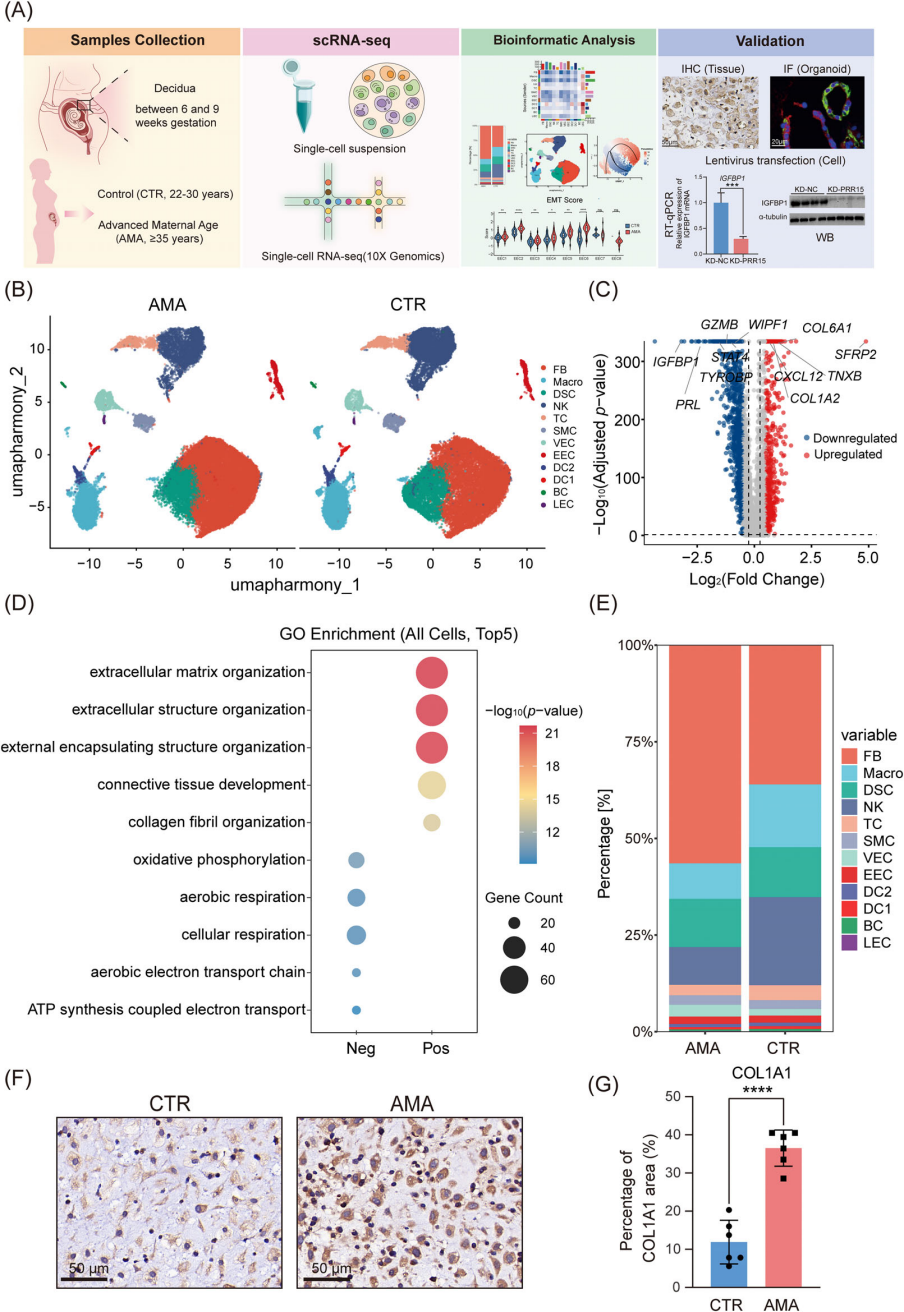
Single‐cell profiling reveals a fibrotic, immunosuppressive AMA decidual microenvironment. (A) Workflow for decidual scRNA‐seq. (B) UMAP visualisation of the 12 identified cell types, split by condition (AMA, left panel; CTR, right panel): fibroblasts (FB), decidual stromal cells (DSC), endometrial epithelial cells (EEC), smooth muscle cells (SMC), vascular endothelial cells (VEC), lymphatic endothelial cells (LEC), macrophages (Macro), NK cells (NK), dendritic cells (DC1/DC2), T cells (TC) and B cells (BC). (C) Volcano plot of global DGE analysis comparing all cells from AMA vs. CTR groups. (D) GO enrichment analysis dot plot for upregulated (Pos) and downregulated (Neg) genes. (E) Relative proportions of cell types in the scRNA‐seq discovery cohort. (*n* = 3 biological replicates per group) (F) Representative IHC for fibroblast marker COL1A1 in the validation cohort. Scale bar: 50 µm. (G) Quantification of COL1A1‐positive area from (F) (*n* = 6 biological replicates per group). Data are presented as the mean ± SD. Statistical significance was determined by a two‐tailed unpaired Student's *t*‐test (****p < 0.0001).

To elucidate the cellular basis of these defects in AMA, we conducted scRNA‐seq on the first trimester of decidual tissues from a discovery cohort of CTR (*n* = 3) and AMA (*n* = 3) individuals (Figure 2A). After quality control and standardisation (Figure S2A), a total of 62 758 high‐quality cells were retained for analysis. Unsupervised clustering identified 12 distinct cell populations (Figure 2B; Figure S2B,C), including six immune cell types (B cells [BC, *MS4A1*⁺*CD79A*⁺], natural killer cells [NK, *NKG7*⁺*KLRC1*⁺], T cells [TC, *CD3D*⁺*CD69*⁺], dendritic cell type 1 [DC1, *CLEC9A*⁺], dendritic cell type 2 [DC2, *CLEC10A*⁺], macrophages [Macro, *CD14*⁺*CD163*⁺]) and six non‐immune cell types (lymphatic endothelial cells [LEC, *CCL21*⁺*STMN1*⁺], endometrial epithelial cells [EEC, *EPCAM*⁺*KRT8*⁺], vascular endothelial cells [VEC, *CD34*⁺*PCDH17*⁺], smooth muscle cells [SMC, *MGP*⁺*RGS5*⁺], decidualised stromal cells [DSC, *IGFBP1*⁺*PRL*⁺], fibroblasts [FB, *LAMA2*⁺*FBLN1*⁺])^12^, ^13^, ^14^, ^15^, ^16^, ^17^ (Figure S2D).

Before examining individual cell types, we first performed a global DGE analysis comparing all cells from the AMA group to the CTR group. This revealed a profound transcriptomic shift, with a substantial number of DEGs (Figure 2C; Table S8). GO and KEGG pathway analysis showed that upregulated genes were significantly enriched in pathways related to extracellular matrix organisation, collagen fibril organisation, and focal adhesion, indicating a dominant tissue remodelling and pro‐fibrotic programme (Figure 2D; Figure S2E; Table S9). Conversely, downregulated genes were predominantly enriched in metabolic pathways, including the significant suppression of oxidative phosphorylation (Figure 2D; Figure S2E; Table S9). This global analysis provides foundational evidence for a dominant pro‐fibrotic program coupled with suppressed metabolic activity in the AMA decidua.

To identify the cellular drivers of this global shift, we analysed the relative proportions of each cell type. This revealed a trend towards fibroblast expansion and a contraction of key immune lineages, notably macrophages and NK cells, in the AMA group (Figure 2E). The absolute cell counts for all cell types and samples are provided in Table S10. Although these compositional shifts did not reach statistical significance in the small discovery cohort (*p *> 0.05, Table S10), likely due to high inter‐individual variability as visualised in the sample‐specific proportion plot (Figure S2F), they provided a strong hypothesis for subsequent validation.

To confirm this finding, we performed IHC and IF analyses in a larger, independent validation cohort (n = 6 per group). This experiment confirmed a robust and statistically significant increase in fibroblast abundance (marked by COL1A1) in the AMA decidua (Figure 2F,G, additional images in Figure S2G). Concurrently, staining for the macrophage marker CD14 and the NK cell marker CD56 confirmed a statistically significant depletion of these immune cell populations in the AMA tissue (Figure S2H–K). Taken together, these data collectively define the AMA decidua as a microenvironment pathologically skewed towards a pro‐fibrotic and immune‐depleted state.

### 3.3 TGF‐β‐driven fibroblast–myofibroblast transition underlies decidual fibrosis in AMA

**FIGURE 3:**
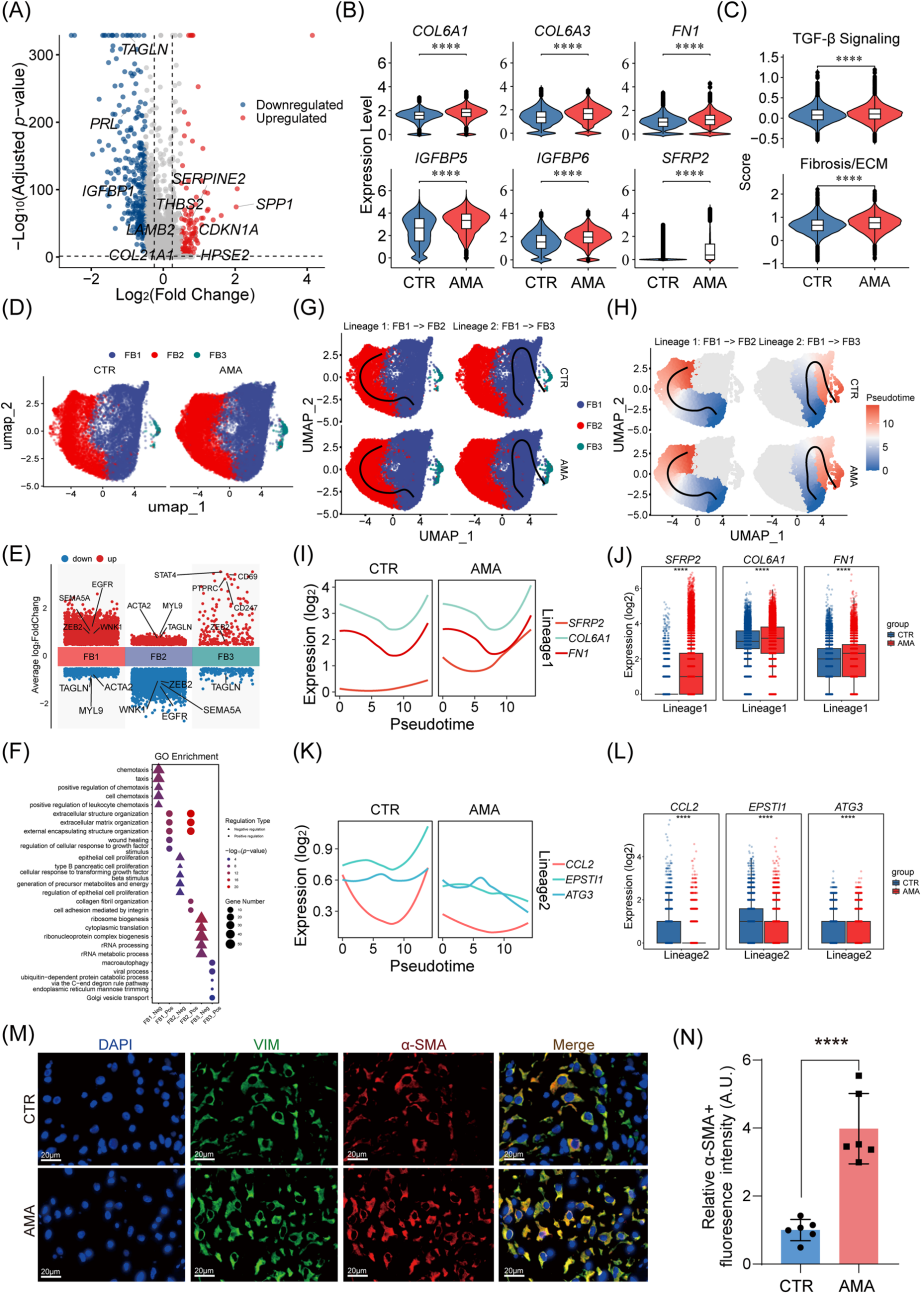
Fibroblast‐to‐myofibroblast transition drives AMA decidual fibrosis. (A) Volcano plot showing global DEGs in all fibroblasts from AMA vs. CTR samples. (B) Violin plots showing upregulation of key pro‐fibrotic genes (*COL6A1*, *COL6A3*, *FN1*, *IGFBP5*, *IGFBP6*, *SFRP2*) in AMA FB. (C) Violin plots showing elevated “TGF‐β Response” and “Fibrosis/ECM Production” scores in AMA FB. (D) UMAP visualisation of three FB subclusters, split by condition (CTR, left; AMA, right). (E) Volcano plots showing top marker genes defining the functional identity of FB1 (progenitor‐like), FB2 (myofibroblast), and FB3 (stress‐responsive) subclusters. (F) Dot plot showing the top 5 enriched GO terms for upregulated (Up) and downregulated (Down) DEGs within each FB subcluster. (G, H) Comparative visualisation of the bifurcated differentiation trajectory, split by condition (CTR vs. AMA). (G) Cells are colored by FB subsets, showing Lineage 1 (FB1→FB2) and Lineage 2 (FB1→FB3). (H) Cells are colored by their inferred pseudotime value (from early/blue to late/red). (I, J) Analysis of pro‐fibrotic gene expression along Lineage 1 (FB1→FB2). (I) Smoothed line plots showing expression trends of *SFRP2*, *COL6A1*, and *FN1* in CTR (left) vs. AMA (right). (J) Binned expression analysis (box plots) confirming significantly higher expression of *SFRP2*, *COL6A1*, and *FN1* in AMA cells. (K, L) Analysis of stress‐response gene expression along Lineage 2 (FB1→FB3). (K) Smoothed line plots showing expression trends of *CCL2*, *EPSTI1*, and *ATG3*. (L) Binned expression analysis (box plots) confirming the failure of robust upregulation of these genes in AMA cells. (M, N) Representative IF images (M) and quantification (N) of α‐SMA (red) and Vimentin (VIM, green) protein, confirming myofibroblast accumulation in AMA decidua (*n* = 6 biological replicates per group). Scale bar: 20 µm. Statistical significance for violin and box plots was determined by a Wilcoxon rank‐sum test. Data in (N) are presented as mean ± SD. Statistical significance was determined by a two‐tailed unpaired Student's *t*‐test (****p < 0.0001).

The AMA decidua exhibited a profound pro‐fibrotic alteration of its FB population. DEG analysis highlighted the significant upregulation of key pro‐fibrotic genes, including the collagen *COL6A1*, the extracellular matrix (ECM) component *SFRP2*, and the TGF‐beta regulator *IGFBP5* (Figure 3A,B; Table S8). Consistent with this, analysis of upregulated genes (Figure S3A, Table S9) confirmed the enrichment of pro‐fibrotic GO pathways, including ‘extracellular matrix organisation’ and ‘extracellular structure organisation’, as well as the KEGG pathway ‘ECM‐receptor interaction’. This transcriptomic shift resulted in significantly elevated ‘TGF‐β response’ and ‘Fibrosis/ECM production’ scores, establishing a pathological activation of AMA fibroblasts (Figure 3C).

To elucidate the cellular origin of decidual fibrosis, we conducted a high‐resolution analysis of 29,029 high‐quality fibroblasts. Unsupervised subclustering delineated three FB subsets (Figure 3D, see integrated and sample‐specific UMAPs in Figure S3B, C). We tested the robustness of the clustering using different resolutions (res = 0.1–0.8) and an alternative (Leiden) algorithm. FB3 was stably identified using both the Louvain algorithm (at res = 0.1) and the Leiden algorithm (at res = 0.1) (Figure S3D). These subsets were annotated based on their unique marker gene expression as: FB1, a progenitor‐like population characterised by markers such as epidermal growth factor receptor (*EGFR*) and *SEMA5A* ^31^; FB2, terminally differentiated, matrix‐producing myofibroblasts expressing *ACTA2* and *MYL9* ^4^, ^32^, ^33^; and FB3, a stress‐responsive subset uniquely expressing immune‐related markers *PTPRC* (*CD45*) and *STAT4* ^34^, ^35^ (Figure 3E).

Although subset proportions revealed no significant differences between the AMA and CTR groups (*p* > 0.05, Figure S3E, Table S10), their intrinsic transcriptional states were profoundly altered in AMA. In AMA, FB1 progenitors suppressed immune chemotaxis programmes (e.g., ‘positive regulation of leukocyte chemotaxis’) (Figure 3F; Table S9) while upregulating structural pathways like ‘ECM‐receptor interaction’ (Figure S3F, Table S9). Concurrently, the FB2 myofibroblast subset exhibited a hyperactive state, strongly upregulating matrix production pathways (e.g., ‘extracellular matrix organisation’) (Figure 3F; Figure S3F; Table S9). The FB3 subset underwent profound metabolic reprogramming, characterised by suppressing core protein synthesis (e.g., ‘ribosome biogenesis’) and upregulating catabolic pathways (e.g., ‘macroautophagy’), consistent with a stressed, dysfunctional state (Figure 3F; Figure S3F; Table S9). Critically, the ‘TGF‐beta signalling pathway’ exhibited complex dysregulation: the ‘TGF‐beta signalling pathway’ was significantly enriched among genes downregulated in AMA (Figure S3F). This observation prompted a more detailed investigation into the pathway's transcriptional state to understand this apparent discrepancy. We therefore quantified the expression of key pathway regulators. The analysis confirmed a systematic downregulation of multiple, distinct inhibitory networks. This included: (1) key intracellular and nuclear negative feedback regulators (*SKI*, *SKIL*, *PPP2CB*, *SMAD6*)^36^, ^37^, ^38^; (2) extracellular antagonists (*BAMBI*, *FST*, *CHRD*)^39^, ^40^, ^41^, ^42^; and (3) the antagonistic, anti‐fibrotic BMP‐ID axis (*BMP2*, *SMAD9*, *ID1*, *ID3*)^43^, ^44^, ^45^ (Figure S3G). The concurrent suppression of these distinct homeostatic networks indicates a pathological failure of self‐regulation. This broad loss of inhibitory controls suggests a mechanism by which the fibroblast population becomes hypersensitive to pro‐fibrotic signalling, thus linking these transcriptional alterations to the overall pro‐fibrotic phenotype.

To model these differentiation dynamics, we performed trajectory inference using the Slingshot, which revealed a bifurcated path from FB1 progenitors to FB2 and FB3 terminal states, where AMA cells preferentially accumulated at these terminal, late‐pseudotime states (Figure 3G,H; Figure S3H,I). Along the FB1‐to‐FB2 (Lineage 1) path, AMA cells showed enhanced matrix‐remodelling activity, with significant upregulation of ECM components *SFRP2*, *COL6A1*, and *FN1* was significantly elevated in the AMA group (Figure 3I,J; Table S11). Concurrently, the FB1‐to‐FB3 (Lineage 2) path revealed a profound failure of functional activation in the AMA group. In CTR cells, this progression was marked by the robust upregulation of key stress‐response and immune‐signalling genes (e.g., *CCL2*, *EPSTI1*, *ATG3*). In contrast, AMA cells failed to mount this adaptive response; *CCL2* expression remained flat at a basal low level, whereas *EPSTI1* and *ATG3* exhibited a declining trend (Figure 3K,L; Table S11). These results provide dynamic molecular evidence that AMA drives a global activation of the FB1 progenitor pool, leading to the increased output of two distinct, pathological cell populations: a hyperactive matrix‐remodelling population (FB2) and a functionally impaired, stress‐response‐deficient population (FB3).

To validate this pro‐fibrotic signature, we confirmed the significant upregulation of *ACTA2* and *COL1A1* in our expanded patient cohort (Figure S3J,K) and observed increased alpha‐smooth muscle actin (α‐SMA) protein deposition (Figure 3M,N). Critically, to functionally prove that this entire process is TGF‐β dependent, we treated primary fibroblasts from AMA donors with a TGF‐β receptor I inhibitor (SB431542).^46^ This treatment completely abrogated the heightened basal expression of myofibroblast markers (*COL1A1*, *ACTA2*) (Figure S3L,M). Collectively, these integrated analyses define a pathological transition of progenitor fibroblasts, resulting in functionally aberrant cell states that collectively promote age‐associated decidual fibrogenesis.

### 3.4 Aberrant fibroblast‐derived TGF‐β signalling drives decidual stromal cell dysfunction in AMA

**FIGURE 4:**
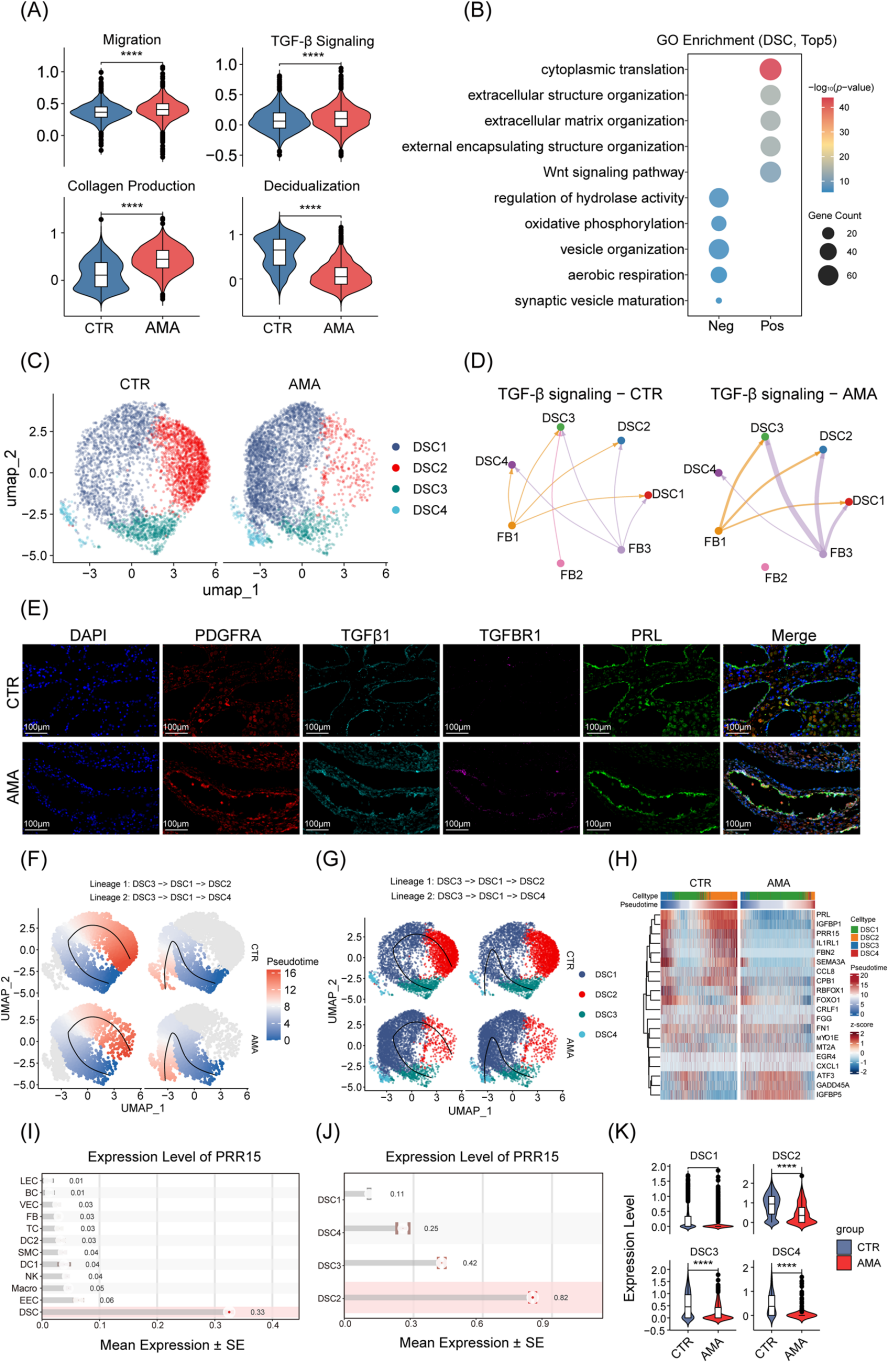
DSC heterogeneity and MET failure in AMA. (A) Pathway activity scores for ‘Migration’, ‘TGF‐β Signalling’, ‘Collagen Production’, and ‘Decidualisation’ in the global DSC population, comparing AMA and CTR groups. Statistical significance was determined by a two‐sided Wilcoxon rank‐sum test. (B) Enriched GO terms (Top 5) for upregulated (Pos) and downregulated (Neg) differentially expressed genes in AMA DSCs compared with CTR. (C) UMAP visualisation of the four DSC subsets (DSC1‐4), split by condition (CTR, left; AMA, right). (D) Chord plot illustrating the enhanced TGF‐β signalling network from FB subsets to DSC subsets in the AMA group compared with CTR. The thickness of the lines represents the communication strength. (E) Representative multiplex immunofluorescence images showing DSCs (PRL, green), FB (PDGFRA, red), TGF‐β1 (cyan), and TGFBR1 (pink) in AMA decidua. Nuclei are stained with DAPI (blue). Scale bar: 20 µm. (*n* = 6 biological replicates per group). (F, G) Comparative visualisation of the bifurcated differentiation trajectory, split by condition (CTR vs. AMA) and colored by pseudotime (F) and cell subtype (G). Lineage 1: DSC3 → DSC1 → DSC2; Lineage 2: DSC3 → DSC1 → DSC4. (H) Comparative pseudotime heatmap, visualising key gene clusters (fibrosis/stress and decidualisation) along the pseudotime axis in CTR and AMA groups. (I) Bar plot showing *PRR15* expression across all 12 identified cell types in decidual tissue. (J) Bar plot showing *PRR15* expression across the four DSC subsets. (K) Violin plot comparing *PRR15* expression in the total DSC population between CTR and AMA groups. Bar plot (I, J) represents mean ± SEM. Violin plot (A, K) statistical significance was determined by a Wilcoxon rank‐sum test (****p < 0.0001).

To investigate how fibroblast‐derived TGF‐β signalling contributes to stromal dysfunction in AMA decidua, we first performed a global transcriptomic comparison of all 7963 DSCs between the AMA and CTR groups. Pathway scoring revealed a profound functional impairment in AMA DSCs, which exhibited significantly elevated activity for ‘migration’, ‘TGF‐β signalling’ and ‘collagen production’ and a concurrently diminished ‘decidualisation’ score (Figure 4A). This was underpinned by the upregulation of key pro‐fibrotic genes (*FN1*, *COL6A1*)^47^ and the cellular stress‐response gene *GADD45A* ^48^ and downregulation of decidualisation effectors (*IGFBP1*, *PAEP, PRL*)^49^ (Figure S4A). GO analysis confirmed this pro‐fibrotic shift, with upregulated genes enriched for ‘cytoplasmic translation’ and ‘extracellular matrix organisation’. Concurrently, downregulated genes enriched for energy production pathways (‘oxidative phosphorylation’ and ‘aerobic respiration’), suggesting metabolic suppression consistent with impaired decidualisation (Figure 4B; Table S9).

To understand the cellular origins of this dysfunction, we resolved the DSCs into four subsets using the Louvain algorithm (res = 0.2) (Figure 4C; see Figure S4B sample‐specific UMAPs and Figure S4C for integrated UMAP). We noted that alternative clustering algorithms (e.g., Leiden) and resolutions did not yield an identical partition (Figure S4D). However, we retained this four‐cluster solution as it provided a clear functional separation that is consistent with known decidual stromal heterogeneity and published literature.^17^, ^31^, ^50^, ^51^ These subsets were annotated based on their marker genes as: an activated, matrix‐producing state (DSC1), marked by matrix‐related genes (*FN1*, *COL3A1*, and *VIM*)^52^; a mature, hormone‐responsive state (DSC2), expressing canonical decidualisation markers (*PRL*, *IGFBP1*, *WNT4*, and *HAND2*)^51^, ^52^; a mesenchymal‐to‐epithelial transition (MET)‐primed state (DSC3), marked by MET‐related genes (*GJA1*, *LAMC1*, and *CDH11*)^53^, ^54^; and a pro‐inflammatory state (DSC4), defined by inflammatory genes (*NFKB1*, *STAT3*, *IL6* and *TNF*)^50^ (Figure S4E; Table S9). Although DSC subset proportions revealed no significant differences between the AMA and CTR groups (Figure S4F; Table S10), cell–cell communication analysis identified a potent external driver: enhanced TGF‐β signalling originating from fibroblasts and targeting the DSC populations in AMA (Figure 4D). We validated this signalling axis in situ, confirming increased TGF‐β ligand intensity in the AMA decidua (Figure 4E).

To map the differentiation dynamics, we performed pseudotime trajectory analysis using Slingshot. Grounded in the biological understanding of decidualisation as a MET,^55^ we defined the MET‐primed and least differentiated DSC3 subset as the trajectory's root state. We reconstructed a physiological differentiation trajectory originating from the MET‐primed DSC3 cluster, which progressed through the intermediate activated DSC1 state before reaching the terminal, mature DSC2 state (Lineage 1: DSC3 → DSC1 → DSC2). A minor pathological branch to the DSC4 state was also identified (Lineage 2: DSC3 → DSC1 → DSC4) (Figure 4F,G; Figure S4G,H). Our primary focus was on the physiological path, Lineage 1, as its disruption directly impacts decidualisation. Critically, a comparative pseudotime heatmap analysis revealed that AMA cells fail to execute this physiological transition (Figure 4H). In CTR cells, the trajectory analysis showed a clear progression of cells to the terminal DSC2 state, which robustly activated the decidualisation program (e.g., *PRL*, *IGFBP1*, *PRR15*). In contrast, AMA cells accumulated at the DSC1 intermediate state, resulting in a significant reduction of terminal DSC2 cells. This differentiation arrest was coupled with a pathological activation of the stagnated DSC1 population, which aberrantly upregulated a battery of pro‐fibrotic and pro‐inflammatory genes, including *GADD45A*, *ATF3*, and the TGF‐β regulator *IGFBP5* (Figure 4H). To dissect the molecular kinetics of this differentiation blockade along Lineage 1, we modelled the gene expression dynamics. The path displayed a ‘V‐shaped’ dynamic, matching the DSC3 → DSC1 (intermediate) → DSC2 (terminal) trajectory. Consistent with impaired maturation, AMA cells exhibited significantly lower baseline and terminal expression levels of the core decidualisation markers *PRL*, *IGFBP1*, and *PRR15* compared with CTR (Figure 4SI,K; Table S11). Consistent with impaired maturation, AMA cells exhibited significantly higher baseline and terminal expression levels of the core pro‐fibrotic markers *GADD45A*, *ATF3*, and *IGFBP5* compared with CTR (Figure S4J,L, Table S11).

Among the genes that failed induction, we focused on *PRR15*, a known regulator of gestational competence.^56^, ^57^, ^58^ Our transcriptomic analysis identified *PRR15* as a key transcript specifically enriched in DSCs among all identified cell types (Figure 4I), particularly in the healthy, mature DSC2 subset (Figure 4J). It was also significantly downregulated in AMA decidua stromal cells (Figure 4K; Table S8). To further solidify its role as a key regulator rather than just a downstream marker like *PRL*, we performed a correlation analysis between *PRR15* expression and the key functional scores across all six samples. This analysis provided a critical quantitative link: *PRR15* mean expression showed a highly significant positive correlation with the ‘decidualisation’ score (Figure S4M, Spearman rho = 0.943, *p* = 0.0167) and a highly significant negative correlation with the ‘collagen production’ score (Figure S4N, Spearman rho = −0.943, *p *= 0.0167, Table S11). This strong quantitative correlation with the core decidual function (decidualisation) and the primary pathology (collagen production), combined with its specific expression profile, strongly supported our hypothesis that PRR15's potential upstream regulatory role in this context is uncharacterised. We therefore hypothesised that this decline is a key mechanistic driver of the observed stromal dysfunction, not merely a consequence, making it a prime candidate for functional investigation.

### 3.5 PRR15 deficiency disrupts decidualisation via uncontrolled TGF‐β signalling

**FIGURE 5:**
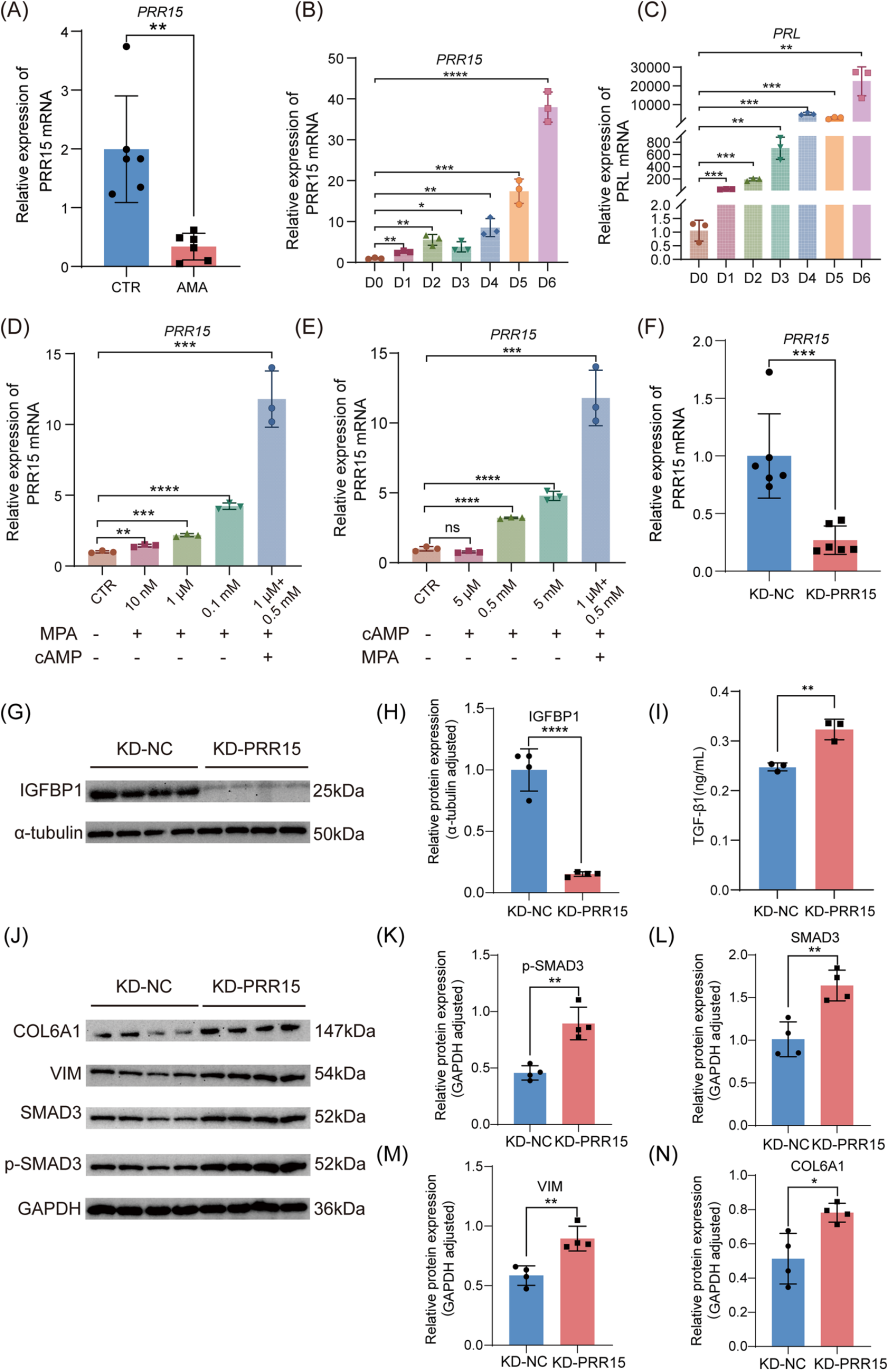
PRR15 is identified as a critical regulator of decidualisation. (A) RT‐qPCR analysis of *PRR15* mRNA expression in decidual tissue from CTR and AMA individuals (*n* = 6 biological replicates per group). (B, C) RT‐qPCR analysis of *PRR15* (B) and *PRL* (C) mRNA expression in primary stromal cells at the indicated time points (days) following initiation of in vitro decidualisation (*n* = 3 biological replicates). (D, E) RT‐qPCR analysis of *PRR15* mRNA expression in stromal cells treated for 4 days with varying concentrations of medroxyprogesterone acetate (MPA) (D) or 8‐bromoadenosine 3′,5′‐cyclic monophosphate (cAMP) (E), alone or in combination (1 µM MPA + 0.5 mM cAMP) (*n* = 3 biological replicates). (F) RT‐qPCR analysis of *PRR15* knockdown (KD‐PRR15) efficiency compared with non‐targeting control (KD‐NC) (*n* = 6 biological replicates). (G, H) Representative Western blot (G) and quantification (H) of the decidualisation marker IGFBP1 in KD‐NC or KD‐PRR15 cells cultured under decidualisation conditions for 4 days (*n* = 4 biological replicates). (I) ELISA of secreted TGF‐β1 protein in the culture medium from cells treated as in (F) (*n* = 3 biological replicates). (J) Representative Western blots of total and phosphorylated SMAD3 (p‐SMAD3), SMAD3, Vimentin (VIM), and COL6A1 in cells treated as in (F). (K–N) Densitometric quantification of the protein bands from the Western blots shown in (J): (K) p‐SMAD3, (L) SMAD3, (M) VIM, and (N) COL6A1 (*n* = 4 biological replicates). Data are presented as mean ± SD. Comparisons were made by two‐tailed unpaired Student's *t*‐test (two groups) or one‐way ANOVA with Tukey's post hoc test (multiple groups) (ns, not significant; *p < 0.05, **p < 0.01, ***p < 0.001, ****p < 0.0001).

To test our hypothesis that the loss of PRR15 is a key mechanistic driver of the observed stromal dysfunction, we next sought to validate its dysregulation in our patient cohort and functionally investigate its role in both physiological decidualisation and the TGF‐β pathway. We first confirmed the significant reduction of *PRR15* mRNA in our expanded AMA cohort (Figure 5A). To establish its physiological relevance to decidualisation, we next examined its expression dynamics during this process. In primary stromal cells undergoing in vitro decidualisation, the expression of *PRR15* was robustly induced over time, mirroring the pattern of the canonical decidual marker *PRL* (Figure 5B, C). This induction was confirmed to be hormone‐dependent, as *PRR15* expression was significantly upregulated by treatment with medroxyprogesterone acetate (MPA) and 8‐bromoadenosine 3′,5′‐cyclic monophosphate (cAMP), particularly when used in combination (Figure 5D,E). These findings collectively indicate that *PRR15* is a hormone‐responsive, decidualisation‐associated gene, suggesting that its loss in AMA could be causally impairing this process.

To directly test this causal role, we performed lentivirus‐mediated knockdown of *PRR15* in primary stromal cells (Figure 5F). Loss of *PRR15* severely impaired decidual competence. Phenotypically, the knockdown cells retained a fibroblastic morphology (Figure S5A) and exhibited a significantly reduced induction of the key decidualisation marker *IGFBP1* (Figure 5G, H), with the most pronounced suppression observed during the early stages of differentiation (Figure S5B). Mechanistically, this decidualisation failure was associated with increased TGF‐β/SMAD signalling. *PRR15* knockdown led to a marked increase in the secretion of the TGF‐β1 ligand (Figure 5I) and a coordinated upregulation of phosphorylated SMAD2 and SMAD3 (p‐SMAD2/3) (Figure 5J–L Figure S5C,D). Consequently, the expression of downstream fibrotic effector proteins, including Vimentin and COL6A1, was also significantly increased (Figure 5J, M, N). To functionally establish the causal link between *PRR15* loss, TGF‐β hyperactivation, and decidualisation failure, we performed a mechanistic rescue experiment. We treated the *PRR15*‐knockdown cells with SB431542, a specific inhibitor of the TGF‐β pathway. Remarkably, this treatment successfully ameliorated the decidualisation defect, as evidenced by the restored secretion of IGFBP1 protein (Figure S5E). Furthermore, immunofluorescence analysis confirmed that SB431542 treatment effectively blocked the PRR15‐knockdown‐induced nuclear translocation of SMAD2/3 (Figure S5F,G).

Conversely, gain‐of‐function experiments solidified *PRR15*’s role as a negative regulator of this pathway. Overexpression of *PRR15* (Figure S5H) not only enhanced the induction of *IGFBP1* (Figure S5I) but also effectively suppressed the pro‐fibrotic signalling cascade, including a reduction in TGF‐β1 secretion (Figure S5J) and downregulation of key fibrotic target genes (Figure S5K,L). Collectively, these loss‐ and gain‐of‐function data define *PRR15* as an essential negative regulator of pro‐fibrotic TGF‐β signalling, the loss of which in the ageing decidua disrupts stromal cell fate and contributes to decidualisation failure.

### 3.6 TGF‐β activation drives EEC epithelial‐to‐mesenchymal transition (EMT) to amplify fibrosis in AMA decidua

**FIGURE 6:**
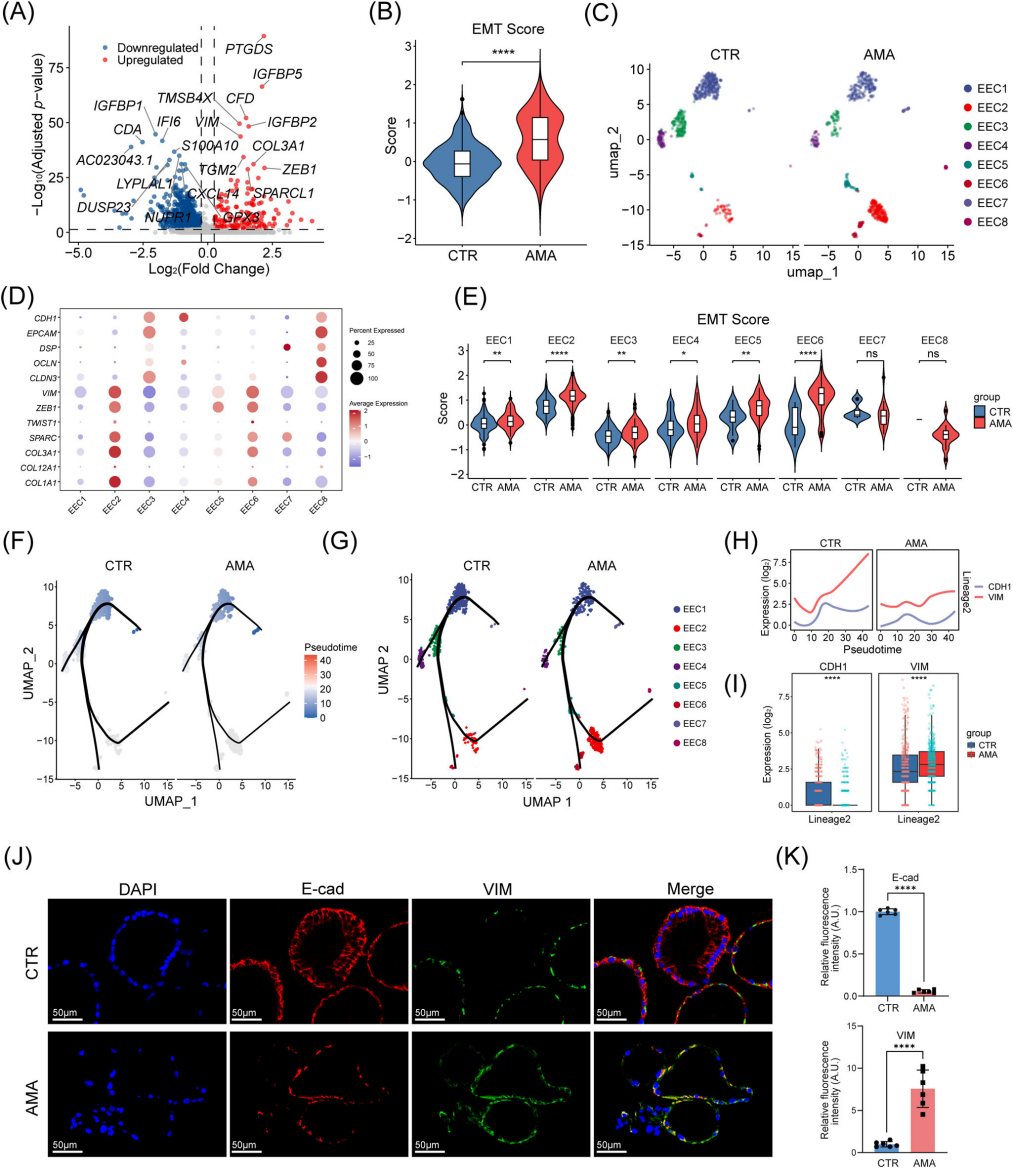
Epithelial–mesenchymal reprogramming of EEC in the AMA decidua. (A) Volcano plot of differentially expressed genes in the global EEC population, comparing AMA versus CTR groups. (B) Violin plot of the pan‐EEC EMT signature score for all EECs from the CTR and AMA groups (*n* = 3 biological replicates). (C) UMAP visualisation of the eight identified EEC subsets, shown split by condition (CTR (left) vs. AMA (right)). (D) Dot plot showing the expression of key epithelial and mesenchymal marker genes across the eight EEC subsets. (E) Violin plot of EMT scores within each EEC subset, comparing CTR and AMA groups. (F, G) Pseudotime trajectory analysis of EEC differentiation, shown split by condition. Panels display the trajectories colored by pseudotime (F) and by cell subset (G). (H) Smoothed line plots comparing the expression dynamics of the epithelial marker *CDH1* and the mesenchymal marker *VIM* along Lineage 2 between CTR and AMA groups. (I) Binned expression analysis (box plots) of *CDH1* and *VIM* along Lineage 2. (J, K) Representative immunofluorescence images (J) of epithelial organoids (EEOs) derived from women of CTR and AMA patients, stained for E‐cadherin (red) and Vimentin (green) and quantification (K) of fluorescence intensity (*n* = 6 biological replicates per group). Nuclei are stained with DAPI (blue). Scale bar: 20 µm. Statistical significance for violin/box plots (B, E, I) was determined by a Wilcoxon rank‐sum test. Bar graphs (K) represent mean ± SD; statistical significance was determined by a two‐tailed unpaired Student's *t*‐test (****p < 0.0001).

Although TGF‐β is a known EMT inducer,^59^ its impact on EEC in decidual fibrosis was unclear. To investigate the epithelial contribution to the pro‐fibrotic microenvironment, we analysed 1,172 high‐quality EECs. Global DEG analysis revealed a transcriptomic shift consistent with EMT in AMA, marked by the coordinate upregulation of the key EMT transcription factor (*ZEB1*) and ECM‐associated genes (*COL3A1*, *SPARCL1*) (Figure 6A; Table S8). GO analysis confirmed enrichment of matrix‐related pathways among upregulated genes (Figure S6A; Table S9). Consistently, a pan‐EEC EMT signature score was significantly elevated in the AMA group (Figure 6B).

Subclustering resolved EECs into eight subsets (Figure 6C; Figure S6B,C). The robustness of this 8‐cluster annotation was confirmed using varied resolutions (res = 0.1– 0.8) and an alternative (Leiden) algorithm, which reproduced the original Louvain (res = 0.5) solution (Figure S6D). The EEC2 subset uniquely expressed high levels of mesenchymal markers (*COL1A1*, *VIM*, *ZEB1*) (Figure 6D). While EEC2 proportions showed a trend towards an increase in AMA, this was not statistically significant (*p* = 0.4, Figure S6E; Table S10), likely due to high inter‐patient variability, suggesting altered cell state rather than expansion drives the EMT signature. Within the EEC2 subset, cells from women of AMA displayed significantly higher per‐cell EMT scores (Figure 6E). To rigorously validate this, we performed a Gene Set Enrichment Analysis (GSEA) comparing AMA vs. CTR cells within the EEC2 subset. This analysis confirmed a powerful and highly significant enrichment for the ‘HALLMARK_EPITHELIAL_MESENCHYMAL_TRANSITION’ pathway (*p*‐value = 10^−10^), providing definitive evidence of an activated EMT state (Figure S6F).

To validate these findings dynamically, we performed pseudotime trajectory analysis using Slingshot, identifying three principal lineages (Figure 6F,G; Figure S6G,H). Lineage 2 represented the primary path towards the EEC2 state (Figure 6G; Figure S6H). However, smoothed expression profiles along Lineage 2 suggested that *VIM* expression might be lower in AMA cells compared to CTR cells at later pseudotime points (Figure 6H). To enable a direct, stage‐matched comparison less susceptible to potential smoothing or cell density biases, we quantified average gene expression within discrete pseudotime bins. This binned analysis revealed significantly lower *CDH1* and significantly higher *VIM* expression in AMA cells compared to CTR cells within equivalent pseudotime intervals along Lineage 2 (Figure 6I; *p* < 0.001, Wilcoxon test; Table S11).

This quantitative trajectory result, indicating an enhanced EMT state in AMA EECs, was consistent with the elevated EMT scores (Figure 6B,E) and validated at the protein level by E‐cadherin loss and Vimentin gain in AMA‐derived organoids (*n* = 6 vs. 6) (Figure 6J,K). Collectively, these convergent data demonstrate that epithelial–mesenchymal reprogramming within EECs, particularly the functional activation and EMT intensification of the TGF‐β‐responsive EEC2 subset, acts as a critical pathogenic amplifier of age‐related fibrogenesis in the AMA decidua.

### 3.7 Immune cells in AMA decidua display a pro‐fibrotic transcriptomic signature

**FIGURE 7:**
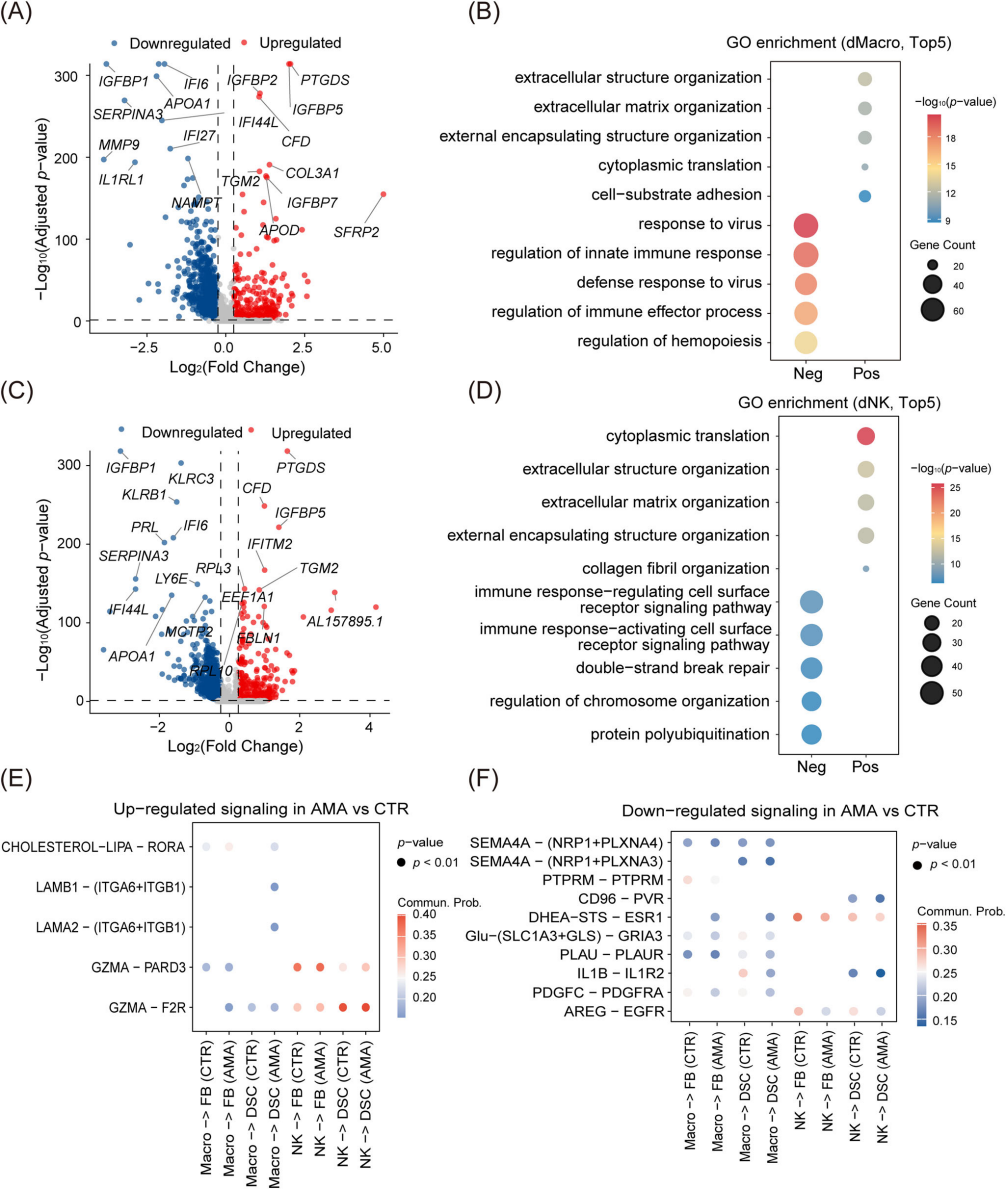
Decidual immune cells in AMA are pro‐fibrotic and impaired. (A) Volcano plot of DEGs in dMacros, comparing AMA versus CTR groups. (B) Enriched GO terms (Top 5) for upregulated (Pos) and downregulated (Neg) DEGs in AMA dMacros. (C) Volcano plot of DEGs in dNK cells, comparing AMA versus CTR groups. (D) Enriched GO terms (Top 5) for upregulated (Pos) and downregulated (Neg) DEGs in AMA dNK cells. (E) Dot plot showing significantly upregulated signalling pathways (*p *< 0.01) originating from dMacros and dNK cells and targeting FB and DSCs in AMA. (F) Dot plot showing significantly downregulated signalling pathways (*p* < 0.01) in AMA.

To investigate whether immune cells also contribute to the fibrotic changes observed in AMA decidua, we performed transcriptional profiling of dMacro and dNK. dMacro displayed a marked transcriptomic shift towards a pro‐fibrotic signature, with upregulation of extracellular matrix (ECM)‐associated genes, including *IGFBP5*, *COL3A1*, and *TGM2* (Figure 7A; Table S8). Pathway analysis supported this shift, showing that upregulated genes were significantly enriched for ‘extracellular matrix organisation’ (Figure 7B) and pro‐fibrotic signalling pathways, such as ‘Focal adhesion’ and ‘ECM‐receptor interaction’ (Figure S7A; Table S9). Concurrently, dMacro downregulated key endometrial function‐related genes, such as *IGFBP1* and *MMP9* (Figure 7A; Table S8), and suppressed key immune surveillance pathways like ‘response to virus’, ‘regulation of innate immune response’ and ‘lysosome’ (Figure 7B; Figure S7A; Table S9). These data suggest a functional shift from an immune surveillance profile towards a matrix‐regulating state. Similarly, dNK cells exhibited a profound loss of their cytotoxic identity in favour of a tissue‐remodelling role. dNK cells exhibited compromised effector profiles with reduced expression of antiviral (e.g., *IFI44L*),^60^ cytotoxic (e.g., *KLRB1*),^61^ and hormone/chemokine‐responsive genes (Figure 7C; Table S8), and suppression of core immune pathways, such as ‘immune response‐regulating cell surface’ (Figure 7D) and ‘dNK‐mediated cytotoxicity’ (Figure S7B; Table S9). In parallel, dNK cells upregulated ECM modulators such as *FBLN1* and *TGM2* (Figure 7C; Table S8), suggesting a change from a cytotoxic profile to roles in tissue remodelling, as supported by the upregulation of ‘extracellular structure organisation’ (Figure 7D) and ‘ECM‐receptor interaction’ (Figure S7B; Table S9).

This collective transcriptional reprogramming establishes decidual immune cells as active contributors to the dysfunctional stromal landscape in the AMA decidua. To determine how this dysregulation impacts the stromal microenvironment, we computationally inferred the cell–cell communication (CCC) landscape. We first analysed signals originating from immune cells and directed to the stroma. This revealed both a gain of pathological signalling and a loss of essential supportive functions in AMA. As shown in Figure 7E, AMA dNK cells significantly upregulated Granzyme A (GZMA) signalling to both FB and DSCs. Extracellular GZMA functions as a protease known to promote inflammation and ECM remodelling by cleaving receptors like F2R (PAR‐1),^62^ suggesting dNKs actively contribute to tissue disruption. Furthermore, AMA macrophages acquired aberrant signalling pathways not present in CTR, including CHOLESTEROL‐LIPA and LAMB1 signalling to DSCs (Figure 7E). The Laminin (LAMB1) signal suggests that macrophages abnormally contribute to ECM deposition,^63^ while the accumulation of the CHOLESTEROL pathway implies a state of pro‐fibrotic activation.^64^ Conversely, immune cells failed to provide key supportive and protective signals. Figure 7F illustrates the significant downregulation of the NK → DSC signal AREG—EGFR. AREG is known to be important for promoting successful decidualisation.^65^ Additionally, the Macro → DSC pathway IL‐1B—IL1R2 was significantly downregulated in AMA (Figure 7F). IL1R2 is a decoy receptor that sequesters pro‐inflammatory IL‐1B.^66^ The loss of this protective pathway indicates a failed mechanism for resolving inflammation, likely leading to amplified IL‐1B‐driven tissue damage.

We further analysed the feedback signals originating from the stroma and directed towards to immune cells. As shown in Figure S7C, we observed a massive upregulation of ECM‐derived signals from FB to immune cells in AMA, dominated by COLLAGEN (e.g., COL1A1, COL6A1) and LAMININ (e.g., LAMA2) pathways. This intense collagen‐integrin signalling is known to modulate immune cell behaviour and drive fibrosis progression,^67^ suggesting that the fibrotic stroma actively reinforces the pro‐fibrotic transcriptional state of resident immune cells. In parallel, Figure S7D reveals a breakdown in immune‐tolerance signalling. The canonical HLA‐E‐(CD94+NKG2E) pathway, a critical mechanism for inhibiting dNK cytotoxicity and maintaining maternal‐fetal tolerance,^68^ was significantly downregulated from DSCs to dNKs in AMA.

### 3.8 Global disruption of cell‐to‐cell communication networks in the AMA decidua

To delineate the systems‐level coordination of the altered cellular landscape, we conducted a comprehensive analysis of the decidual intercellular communication network. This revealed a global reduction in both the total number of inferred interactions and the cumulative interaction strength within the AMA decidua, indicating a significant attenuation of overall network connectivity (Figure S8A,B). By dissecting this network‐wide disruption, we uncovered multi‐faceted dysregulation across distinct biological axes crucial for pregnancy.

**FIGURE 8:**
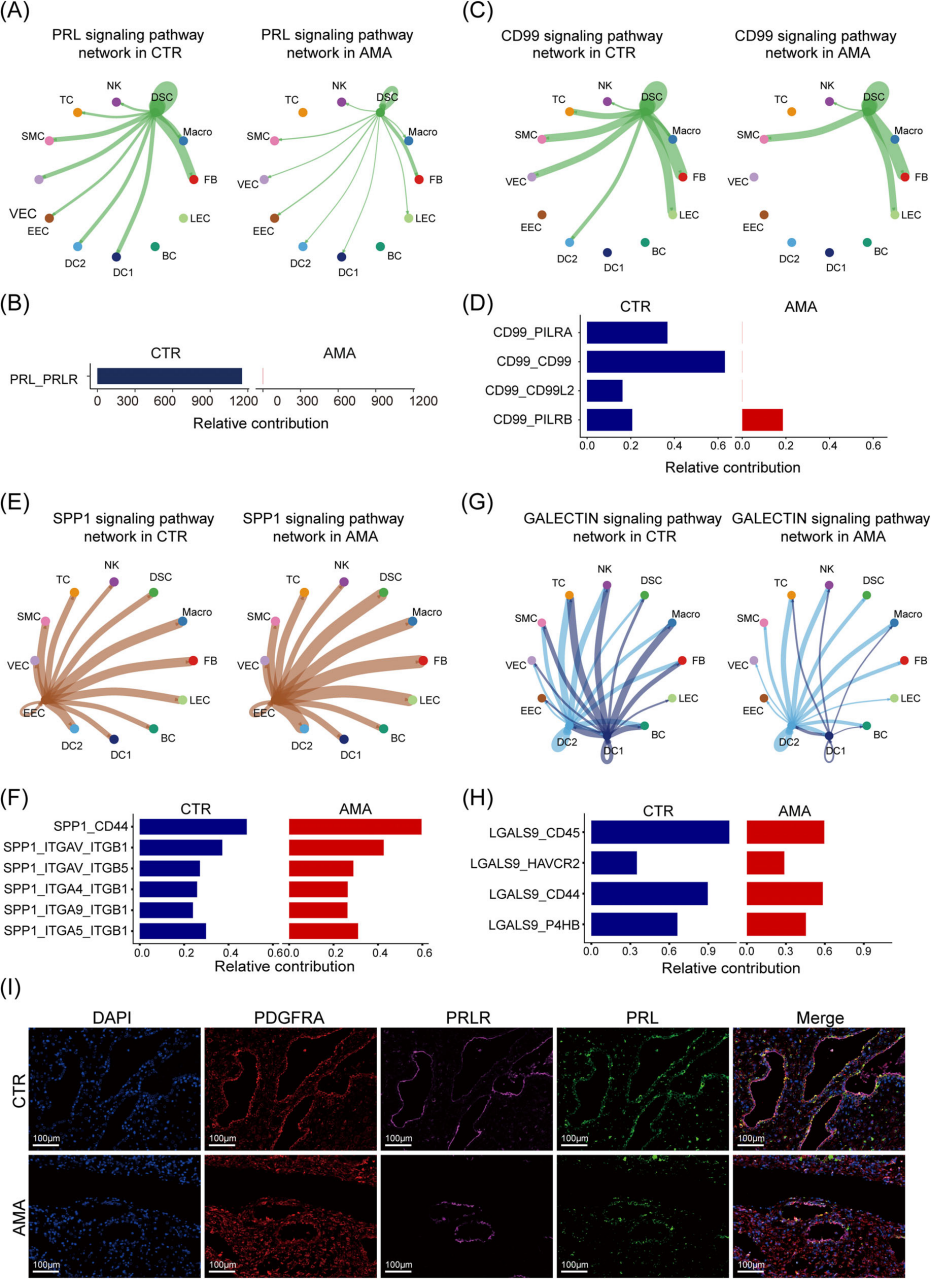
Dysregulated cell‐to‐cell communications in AMA. (A, B) Chord plots (A) and relative contribution analysis (B) of the PRL signalling pathway. (C, D) Chord plots (C) and relative contribution analysis (D) of the CD99 signalling pathway. (E, F) Chord plots (E) and relative contribution analysis (F) of the SPP1 signalling pathway. (G, H) Chord plots (G) and relative contribution analysis (H) of the GALECTIN signalling pathway. (I) Representative multiplex immunofluorescence images showing reduced PRL (green) and PRLR (magenta) expression co‐localised with FB (PDGFRA, red) in AMA tissue. Nuclei are stained with DAPI (blue). Scale bar: 100 µm. (*n* = 6 biological replicates).

First, pathways essential for establishing and maintaining decidual homeostasis were severely compromised. As a cornerstone of decidualisation, PRL signalling originating from DSCs was markedly diminished in AMA (Figure 8A,B). This fundamental failure was compounded by the attenuation of the epidermal growth factor (EGF) family of pro‐regenerative signals (e.g., AREG, HBEGF) (Figure S8C,D) and weakened wingless/integrated (WNT) signalling (Figure S8E,F), which are critical for decidual growth and vascular support,^69^ respectively. Immunofluorescence staining confirmed this finding at the protein level, showing markedly reduced expression of both PRL and its receptor, PRLR, in AMA tissue (Figure 8I).

Concurrently, the communication network was rewired to promote a pro‐fibrotic, uncoordinated remodelling state. We observed opposing regulation of the midkine family, with pleiotrophin (PTN) signalling (e.g., PTN‐NCL) enhanced while MDK signalling was attenuated (Figure S8G–J). Compounding this, CD99 signalling, which is critical for endothelial integrity,^70^ was significantly reduced in AMA (Figure 8C,D). Most notably, the pro‐fibrotic osteopontin (SPP1) signalling axis,^71^ predominantly driven by EECs and dMacros, was significantly hyperactivated in AMA (Figure 8E,F). This pathway is a known powerful driver of tissue fibrosis.

**FIGURE 9:**
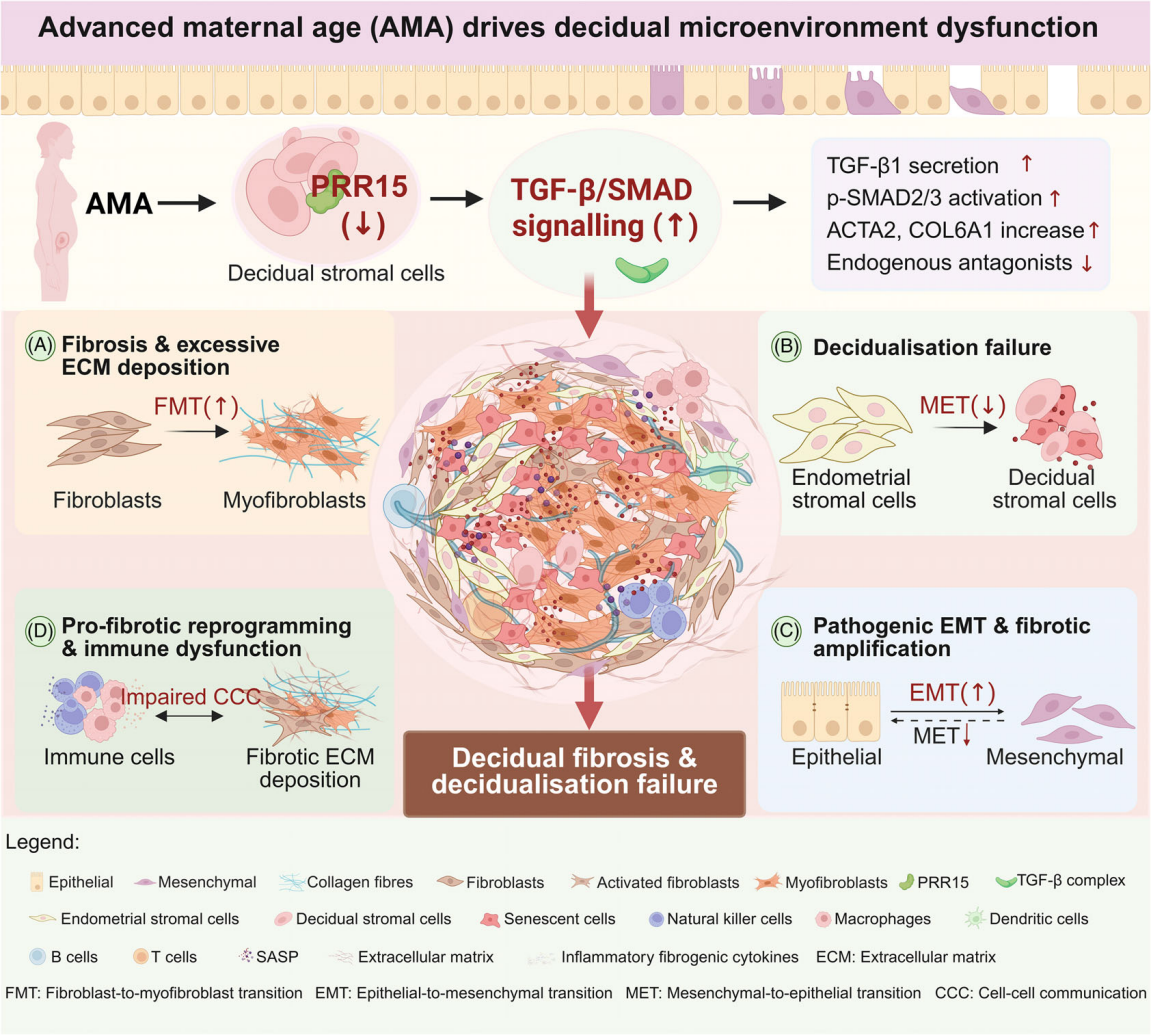
Schematic of the proposed pathophysiology driven by the PRR15‐TGF‐β axis in the AMA decidua. AMA downregulates PRR15 in decidual stromal cells, amplifying TGF‐β/SMAD signalling. This central dysregulation drives four key pathologies: (A) enhanced fibroblast‐to‐myofibroblast transition (FMT), leading to fibrosis; (B) failed mesenchymal‐to‐epithelial transition (MET), causing decidualisation failure; (C) pathogenic epithelial‐to‐mesenchymal transition (EMT) in epithelial cells; and (D) pro‐fibrotic reprogramming of immune cells. Collectively, this PRR15‐TGF‐β axis dysregulation results in decidual fibrosis and decidualisation failure.

Finally, networks that coordinate local immune tolerance were broadly disrupted. Key immunomodulatory pathways, including GALECTIN (e.g., LGALS9)^16^ and CLEC signalling,^72^ were significantly downregulated in AMA (Figure 8G,H; Figure S8K,L), suggesting a failure in immune coordination and tolerance. This was further evidenced by the attenuation of ApoA‐mediated immunomodulatory^73^ signalling from macrophages (Figure S8M,N). Collectively, this dysregulated signalling landscape establishes a mechanistic basis that links individual cellular alterations to the overall decidual dysfunction, characterised by failed homeostasis, pro‐fibrotic remodelling and profound immune dysregulation (Figure 9).

## 4 DISCUSSION

AMA has been primarily linked to adverse pregnancy outcomes due to declining oocyte quality.^74^, ^75^, ^76^ However, successful implantation and pregnancy critically depend on a receptive maternal uterine environment, particularly the decidua.^12^, ^77^, ^78^ Our study provides the first single‐cell transcriptomic atlas of the human decidua in women of AMA, fundamentally shifting the research paradigm from an oocyte‐centric view towards the indispensable contributions of the disturbed decidual microenvironment to age‐related reproductive decline.^75^, ^79^, ^80^ We demonstrated that decidual dysfunction in AMA is not merely a passive failure but an active pathological process driven by pervasive fibrotic remodelling, impaired stromal cell differentiation and a pro‐fibrotic transcriptional shift in local immune populations. This comprehensive single‐cell resolution reveals a far more complex aetiology of age‐related reproductive issues than previously understood, specifically highlighting the compromised decidua microenvironment.^81^, ^82^

A pivotal and novel finding of our research is the identification of the PRR15‐TGF‐β signalling axis as a central mechanistic pathway regulating DSC fate, shifting the focus from well‐established downstream functional markers (such as PRL) to a novel upstream driver of decidual failure. We found that PRR15 expression was significantly downregulated in AMA decidua, particularly in mature DSCs (DSC2 population). In vitro, PRR15 expression mirrored that of canonical decidualisation markers, and its functional knockdown severely impaired decidual transformation, a finding confirmed by functional knockdown, overexpression and chemical inhibition rescue assays. Importantly, our data revealed that PRR15 unleashes pathological TGF‐β/SMAD activation, evidenced by increased TGF‐β1 ligand secretion and SMAD2/3 phosphorylation, which subsequently drives the observed fibrotic remodelling, impaired MET, and ultimately, decidual failure. While TGF‐β‐driven fibroblast‐to‐myofibroblast transition is a well‐established mechanism in various fibrotic diseases,^82^, ^83^, ^84^ its specific and prominent role in the compromised AMA decidua, regulated upstream by PRR15, represents a previously underappreciated and critical pathological feature in uterine ageing. PRR15 has been linked to early pregnancy and trophoblast function,^56^, ^57^, ^58^ but its pivotal role in the integrity of the AMA decidua and its direct mechanistic link to the TGF‐β pathway in this context constitute a novel discovery.

Beyond the PRR15‐TGF‐β axis, our study delved into the multi‐cellular pathogenesis underpinning the pervasive decidual fibrosis observed in AMA. Crucially, our analysis suggests this fibrotic phenotype is not driven by a simple expansion of specific cell populations, but rather by a profound pathological transcriptional shift in their intrinsic cellular state. For instance, while the proportion of terminal myofibroblasts (FB2) did not significantly change in our scRNA‐seq data, the overall fibroblast pool in AMA decidua showed transcriptional signatures of markedly heightened TGF‐β pathway activity. Our findings point to a more intricate mechanism: the AMA fibroblast population exhibited transcriptional suppression of its own endogenous negative feedback regulators, such as *CHRD* and *BAMBI*. This transcriptional profile suggests a loss of intrinsic feedback inhibition, which may render the entire fibroblast population more responsive to pro‐fibrotic ligands, thereby contributing to a self‐sustaining pathological state.^41^, ^85^ Furthermore, we provided evidence that EEC actively undergo EMT; although the proportion of the mesenchymal‐like EEC2 subpopulation also did not significantly increase, these cells adopted a pathogenic state, exhibiting significantly enriched EMT pathway activity and high expression of mesenchymal markers (e.g., *COL3A1*, *VIM* and *ZEB1*), as confirmed using GSEA and binned pseudotime analysis. This dual contribution from both the stromal and epithelial compartments at a molecular level highlights a more intricate aetiology of decidual fibrosis in AMA than was previously understood^7^, ^86^, ^87^

The immune microenvironment of the AMA decidua also underwent a critical transformation. Unlike their typical roles in immune suppression and vascular remodelling,^88^ AMA dMacros and dNK cells in our study upregulated genes associated with ECM remodelling and pro‐fibrotic pathways (e.g., *COL3A1*, *TGM2* and *IGFBP5*). This suggests a functional skewing away from canonical immune surveillance and tolerance roles towards transcriptional programmes involved in tissue matrix regulation. This shift appears reinforced by a pathological feedback loop identified through cell‐cell communication analysis, wherein the fibrotic stroma directs immune cells via COLLAGEN‐based signals, and immune cells, in turn, may propagate tissue disruption through pathways such as GZMA signalling. This finding suggests that these immune cells are active transcriptional contributors to pathological matrix deposition in the ageing decidua, which extends previous observations of systemic ageing and inflammation to localised decidual pathology.^89^

In AMA‐associated decidualisation failure, there is profound dysregulation of DSC plasticity. Decidualisation is a specialised differentiation process involving MET that is vital for a successful pregnancy.^50^, ^90^ Our trajectory analyses indicate that AMA induces a differentiation blockade. Specifically, AMA DSCs fail to progress from the MET‐primed DSC3 state through the intermediate DSC1 state, becoming arrested before reaching the mature, hormone‐responsive DSC2 terminal state. This arrest, rather than a simple loss of function, appears to be the critical pathology. The stagnated DSC1 intermediate population, which under physiological conditions would transition to DSC2, instead acquires an aberrant, stress‐induced transcriptomic profile, pathologically upregulating matrix and stress‐response genes.

Underpinning these individual cellular defects was a global reduction in the complexity of the intercellular communication network. The overall number and strength of inferred interactions were markedly attenuated in AMA decidua, a hallmark feature of ageing tissues.^91^ This network degradation was not random; rather, it involved the systematic failure of pathways essential for decidual health, coupled with the pathogenic amplification of pro‐fibrotic signalling. Crucial pathways for decidualisation (PRL, WNT, EGF),^65^, ^69^ pro‐fibrotic (SPP1),^71^ immune modulation (GALECTIN, CLEC),^16^, ^72^ and endothelial integrity (CD99)^70^ were profoundly dysregulated. This widespread network degradation likely amplifies individual cellular dysfunction, further impairing the coordinated responses necessary for successful pregnancy. This observation resonates with broader ageing research, indicating that compromised cellular communication networks contribute to age‐related tissue and organ dysfunction with age.^91^

Our study has several strengths. First, it provided the first single‐cell transcriptomic atlas of the human decidua in AMA, revealing cell‐type‐specific alterations in a previously uncharacterised and clinically underserved population. Second, we identified and defined the PRR15‐TGF‐β signalling axis as a novel mechanistic pathway regulating decidualisation. Third, these findings highlight the decidua‐specific pathophysiology in women of AMA, demonstrating that fibrotic remodelling and stromal dysfunction emerge as early as the first trimester, shifting the focus from well‐studied oocyte and embryo factors to maternal uterine contributions.

However, our study also has some limitations. First, our initial scRNA‐seq discovery cohort was limited to three individuals per group. While our key findings, such as fibroblast transcriptional changes and immune cell depletion, were subsequently validated in a larger, independent cohort (*n* = 6 per group), the small discovery cohort size limits the statistical power for detecting changes in rare cell populations and may be susceptible to high inter‐individual variability. Second, our clinical cohorts had a relatively narrow range of body mass index and included patients with prior miscarriages (Tables S1 and S2). Future studies with larger, more diverse cohorts are required to disentangle the effects of ageing from these potential confounding variables and strengthen clinical generalizability. Third, while our functional experiments strongly support the PRR15‐TGF‐β axis, the knockdown validation relied on shRNA. Future studies using CRISPR‐Cas9‐mediated knockout would be necessary to definitively confirm target specificity. Moreover, the precise molecular interaction linking PRR15 loss to SMAD phosphorylation, whether it is direct or indirect, remains to be elucidated using techniques such as co‐immunoprecipitation or promoter assays. Fourth, our assertion that immune cells (dMacro, dNK) acquire a pro‐fibrotic function is based on compelling transcriptomic inference (e.g., upregulation of *COL3A1*, *TGM2*); however, this has not yet been validated with functional assays. Direct in vitro co‐culture experiments to measure matrix deposition or cytokine secretion are required to confirm this inferred phenotype. Finally, our analysis of CCC, while informative, is based on transcriptomic inference and lacks spatial context. Integrating spatial transcriptomics or multiplex immunofluorescence would be necessary to confirm the spatial proximity and co‐localisation of the inferred ligand–receptor pairs in situ.

In conclusion, this study provides a comprehensive molecular and cellular atlas of the dysfunctional decidual landscape in women with AMA, critically identifying a novel PRR15‐TGF‐β signalling axis as a key regulator of decidualisation. Our work re‐emphasises the crucial contribution of the decidual environment to age‐related reproductive decline and offers a new scientific foundation for developing multi‐targeted interventions to improve decidual health and pregnancy success in women of AMA.

## AUTHOR CONTRIBUTIONS

Lu Li, Xiaohui Fan and Songying Zhang conceived the study and participated in the overall design, supervision and coordination of the study. Hongliang Xie and Lu Li designed and performed most experiments, data analysis, prepared figures and tables and drafted the manuscript. Yu Lu, Anqi Zheng and Baofeng Rao participated in cellular biological experiments. Aolin Zhang and Cuiyu Yang contributed to clinical sample and data collection and assisted in obtaining ethical approval. Anyao Li participated in scRNA‐seq data analysis. Wenbo Guo contributed to providing constructional suggestions for the in vitro validation experiment. Linhua Hu, Xiaoling Huang, Hongliang Xie and Aolin Zhang participated in clinical sample collection. Chi Chiu Wang revised the manuscript and contributed constructive suggestions for revision. Songying Zhang, Xiaohui Fan and Lu Li supervised this work and revised the manuscript. All authors reviewed and approved the final manuscript.

## CONFLICT OF INTEREST STATEMENT

The authors declare no conflict of interest.

## FUNDING INFORMATION

The project was supported by the Zhejiang Province Traditional Chinese Medicine Science and Technology Project (GZY‐ZJKJ‐24076, Lu Li), High‐level Talents Special Support Program of Zhejiang Province (2024‐KYY‐GXJS‐0026, Lu Li), National Key Research and Development Program of China(2023YFC3504600, Lu Li), ‘Pioneer’ R&D Program of Zhejiang (2024C03106, Xiaohui Fan), Starlit South Lake Leading Elite Program (2023C303007, Xiaohui Fan), Theme‐based Research Scheme, Research Grants Committee (T13‐602/21‐N, Chi Chiu Wang), the Health and Medicinal Research Fund from Health Burden, HKSAR (15160971, Chi Chiu Wang), the “Pioneer” and “Leading Goose” R&D Program of Zhejiang (2025C02123, Cuiyu Yang) and Transverse Research Project of Zhejiang University (2023‐KYY‐A070350007, Lu Li).

## ETHICS STATEMENT

The Ethics Committee of Sir Run Run Shaw Hospital, Zhejiang University School of Medicine, approved this study (approval no.: 2024 Research Ethics Review No. 0761).

## Supporting information



Supporting Information

Supporting Information

Supporting Information

Supporting Information

Supporting Information

Supporting Information

Supporting Information

Supporting Information

Supporting Information

Supporting Information

Supporting Information

Supporting Information

Supporting Information

Supporting Information

## Data Availability

All data supporting the findings of this study are available within the article, its supplementary materials, or from the corresponding author upon request. The raw single‐cell sequence data reported in this paper have been deposited in NCBI's Gene Expression Omnibus (Edgar et al., 2002) and are accessible through GEO Series accession number GSE305088 (https://www.ncbi.nlm.nih.gov/geo/query/acc.cgi?acc%20=%20GSE305088).

## References

[1] Frick AP . Advanced maternal age and adverse pregnancy outcomes. Best Pract Res Clin Obstet Gynaecol. 2021;70:92‐100. doi:10.1016/j.bpobgyn.2020.07.005 32741623

[2] Navot D , Bergh PA , Williams MA , et al. Poor oocyte quality rather than implantation failure as a cause of age‐related decline in female fertility. Lancet. 1991;337(8754):1375‐1377. doi:10.1016/0140-6736(91)93060-m 1674764

[3] Silvestris E , Petracca EA , Mongelli M , et al. Pregnancy by oocyte donation: reviewing fetal‐maternal risks and complications. Int J Mol Sci. 2023;24(18):13945. doi:10.3390/ijms241813945 37762248 PMC10530596

[4] Younesi FS , Son DO , Firmino J , Hinz B . Myofibroblast markers and microscopy detection methods in cell culture and histology. Methods Mol Biol. 2021;2299:17‐47. doi:10.1007/978-1-0716-1382-5_2 34028733

[5] Le Ray C , Scherier S , Anselem O , et al. Association between oocyte donation and maternal and perinatal outcomes in women aged 43 years or older. Hum Reprod. 2012;27(3):896‐901. doi:10.1093/humrep/der463 22252087

[6] Tinelli A , Andjić M , Morciano A , et al. Uterine aging and reproduction: dealing with a puzzle biologic topic. Int J Mol Sci. 2023;25(1):322. doi:10.3390/ijms25010322 38203493 PMC10778867

[7] Pathare ADS , Loid M , Saare M , et al. Endometrial receptivity in women of advanced age: an underrated factor in infertility. Hum Reprod Update. 2023;29(6):773‐793. doi:10.1093/humupd/dmad019 37468438 PMC10628506

[8] Ng SW , Norwitz GA , Pavlicev M , Tilburgs T , Simón C , Norwitz ER . Endometrial decidualization: the primary driver of pregnancy health. Int J Mol Sci. 2020;21(11):4092. doi:10.3390/ijms21114092 32521725 PMC7312091

[9] Gellersen B , Brosens JJ . Cyclic decidualization of the human endometrium in reproductive health and failure. Endocr Rev. 2014;35(6):851‐905. doi:10.1210/er.2014-1045 25141152

[10] Marti‐Garcia D , Martinez‐Martinez A , Sanz FJ , et al. Age‐related uterine changes and its association with poor reproductive outcomes: a systematic review and meta‐analysis. Reprod Biol Endocrinol. 2024;22(1):152. doi:10.1186/s12958-024-01323-6 39616336 PMC11607893

[11] Lucas ES , Vrljicak P , Muter J , et al. Recurrent pregnancy loss is associated with a pro‐senescent decidual response during the peri‐implantation window. Commun Biol. 2020;3(1):37. doi:10.1038/s42003-020-0763-1 31965050 PMC6972755

[12] Suryawanshi H , Morozov P , Straus A , et al. A single‐cell survey of the human first‐trimester placenta and decidua. Sci Adv. 2018;4(10):eaau4788. doi:10.1126/sciadv.aau4788 30402542 PMC6209386

[13] Wang F , Jia W , Fan M , et al. Single‐cell immune landscape of human recurrent miscarriage. Genom Proteom Bioinform. 2021;19(2):208‐222. doi:10.1016/j.gpb.2020.11.002 PMC8602400 33482359

[14] Lu C , Gao R , Qing P , et al. Single‐cell transcriptome analyses reveal disturbed decidual homoeostasis in obstetric antiphospholipid syndrome. Ann Rheum Dis. 2024;83(5):624‐637. doi:10.1136/ard-2023-224855 38331588

[15] Bao S , Chen Z , Qin D , et al. Single‐cell profiling reveals mechanisms of uncontrolled inflammation and glycolysis in decidual stromal cell subtypes in recurrent miscarriage. Hum Reprod. 2023;38(1):57‐74. doi:10.1093/humrep/deac240 36355621

[16] Li Y , Sang Y , Chang Y , et al. A Galectin‐9‐driven CD11c^high^ decidual macrophage subset suppresses uterine vascular remodeling in preeclampsia. Circulation. 2024;149(21):1670‐1688. doi:10.1161/CIRCULATIONAHA.123.064391 38314577

[17] Vento‐Tormo R , Efremova M , Botting RA , et al. Single‐cell reconstruction of the early maternal‐fetal interface in humans. Nature. 2018;563(7731):347‐353. doi:10.1038/s41586-018-0698-6 30429548 PMC7612850

[18] Turco MY , Gardner L , Hughes J , et al. Long‐term, hormone‐responsive organoid cultures of human endometrium in a chemically defined medium. Nat Cell Biol. 2017;19(5):568‐577. doi:10.1038/ncb3516 28394884 PMC5410172

[19] Deryabin PI , Borodkina AV . Stromal cell senescence contributes to impaired endometrial decidualization and defective interaction with trophoblast cells. Hum Reprod. 2022;37(7):1505‐1524. doi:10.1093/humrep/deac112 35604371

[20] Hao Y , Hao S , Andersen‐Nissen E . Integrated analysis of multimodal single‐cell data. Cell. 2021;184:3573‐3587. doi:10.1016/j.cell.2021.04.048 34062119 PMC8238499

[21] McGinnis CS , Murrow LM , Gartner ZJ . DoubletFinder: doublet detection in single‐cell RNA sequencing data using artificial nearest neighbors. Cell Syst. 2019;8(4):329‐337. doi:10.1016/j.cels.2019.03.003 30954475 PMC6853612

[22] Korsunsky I , Millard N , Fan J , et al. Fast, sensitive and accurate integration of single‐cell data with harmony. Nat Methods. 2019;16(12):1289‐1296. doi:10.1038/s41592-019-0619-0 31740819 PMC6884693

[23] Traag VA , Waltman L , van Eck NJ . From Louvain to Leiden: guaranteeing well‐connected communities. Sci Rep. 2019;9(1):5233. doi:10.1038/s41598-019-41695-z 30914743 PMC6435756

[24] Ma S , Ji Z , Zhang B , et al. Spatial transcriptomic landscape unveils immunoglobin‐associated senescence as a hallmark of aging. Cell. 2024;187(24):7025‐7044. doi:10.1016/j.cell.2024.10.019 39500323

[25] Kanke M , Kennedy Ng MM , Connelly S , et al. Single‐cell analysis reveals unexpected cellular changes and transposon expression signatures in the colonic epithelium of treatment‐naïve adult Crohn's disease patients. Cell Mol Gastroenterol Hepatol. 2022;13(6):1717‐1740. doi:10.1016/j.jcmgh.2022.02.005 35158099 PMC9046244

[26] Briggs EM , Rojas F , McCulloch R , Matthews KR , Otto TD . Single‐cell transcriptomic analysis of bloodstream Trypanosoma brucei reconstructs cell cycle progression and developmental quorum sensing. Nat Commun. 2021;12(1):5268. doi:10.1038/s41467-021-25607-2 34489460 PMC8421343

[27] Wu T , Hu E , Xu S , et al. clusterProfiler 4.0: a universal enrichment tool for interpreting omics data. Innovation (Camb). 2021;2(3):100141. doi:10.1016/j.xinn.2021.100141 34557778 PMC8454663

[28] Hao Y , Stuart T , Kowalski MH , et al. Dictionary learning for integrative, multimodal and scalable single‐cell analysis. Nat Biotechnol. 2024;42(2):293‐304. doi:10.1038/s41587-023-01767-y 37231261 PMC10928517

[29] Street K , Risso D , Fletcher RB , et al. Slingshot: cell lineage and pseudotime inference for single‐cell transcriptomics. BMC Genom. 2018;19(1):477. doi:10.1186/s12864-018-4772-0 PMC6007078 29914354

[30] Jin S , Guerrero‐Juarez CF , Zhang L , et al. Inference and analysis of cell‐cell communication using CellChat. Nat Commun. 2021;12(1):1088. doi:10.1038/s41467-021-21246-9 33597522 PMC7889871

[31] Garcia‐Alonso L , Handfield LF , Roberts K , et al. Mapping the temporal and spatial dynamics of the human endometrium in vivo and in vitro. Nat Genet. 2021;53(12):1698‐1711. doi:10.1038/s41588-021-00972-2 34857954 PMC8648563

[32] Afzal J , Du W , Novin A , et al. Paracrine HB‐EGF signaling reduce enhanced contractile and energetic state of activated decidual fibroblasts by rebalancing SRF‐MRTF‐TCF transcriptional axis. Front Cell Dev Biol. 2022;10:927631. doi:10.3389/fcell.2022.927631 36147738 PMC9485834

[33] Oishi M , Shinjo K , Takanari K , et al. Exclusive expression of KANK4 promotes myofibroblast mobility in keloid tissues. Sci Rep. 2024;14(1):8725. doi:10.1038/s41598-024-59293-z 38622256 PMC11018845

[34] Tchou J , Zhang PJ , Bi Y , et al. Fibroblast activation protein expression by stromal cells and tumor‐associated macrophages in human breast cancer. Hum Pathol. 2013;44(11):2549‐2557. doi:10.1016/j.humpath.2013.06.016 24074532 PMC4283499

[35] Nguyen HN , Noss EH , Mizoguchi F , et al. Autocrine loop involving IL‐6 family member LIF, LIF receptor, and STAT4 drives sustained fibroblast production of inflammatory mediators. Immunity. 2017;46(2):220‐232. doi:10.1016/j.immuni.2017.01.004 28228280 PMC5567864

[36] Deheuninck J , Luo K . Ski and SnoN, potent negative regulators of TGF‐beta signaling. Cell Res. 2009;19(1):47‐57. doi:10.1038/cr.2008.324 19114989 PMC3103856

[37] Liu T , Feng XH . Regulation of TGF‐β signaling by protein phosphatases. Biochem J. 2010;430(2):191‐198. doi:10.1042/BJ20100427 20704570 PMC3154754

[38] Itoh S , ten Dijke P . Negative regulation of TGF‐beta receptor/Smad signal transduction. Curr Opin Cell Biol. 2007;19(2):176‐184. doi:10.1016/j.ceb.2007.02.015 17317136

[39] Lin L , Wang Y , Liu W , Huang Y . BAMBI inhibits skin fibrosis in keloid through suppressing TGF‐β1‐induced hypernomic fibroblast cell proliferation and excessive accumulation of collagen I. Int J Clin Exp Med. 2015;8(8):13227‐13234. 26550247 PMC4612932

[40] Hu HH , Chen DQ , Wang YN , et al. New insights into TGF‐β/Smad signaling in tissue fibrosis. Chem Biol Interact. 2018;292:76‐83. doi:10.1016/j.cbi.2018.07.008 30017632

[41] Shangguan L , Ti X , Krause U , et al. Inhibition of TGF‐β/Smad signaling by BAMBI blocks differentiation of human mesenchymal stem cells to carcinoma‐associated fibroblasts and abolishes their protumor effects. Stem Cells. 2012;30(12):2810‐2819. doi:10.1002/stem.1251 23034983

[42] Kristensen SG , Andersen K , Clement CA , Franks S , Hardy K , Andersen CY . Expression of TGF‐beta superfamily growth factors, their receptors, the associated SMADs and antagonists in five isolated size‐matched populations of pre‐antral follicles from normal human ovaries. Mol Hum Reprod. 2014;20(4):293‐308. doi:10.1093/molehr/gat089 24270394

[43] Wang RN , Green J , Wang Z , et al. Bone morphogenetic protein (BMP) signaling in development and human diseases. Genes Dis. 2014;1(1):87‐105. doi:10.1016/j.gendis.2014.07.005 25401122 PMC4232216

[44] Ye Q , Taleb SJ , Zhao J , Zhao Y , Emerging role of BMPs/BMPR2 signaling pathway in treatment for pulmonary fibrosis. Biomed Pharmacother. 2024;178:117178. doi:10.1016/j.biopha.2024.117178 39142248 PMC11364484

[45] Yang J , Li X , Li Y , et al. Id proteins are critical downstream effectors of BMP signaling in human pulmonary arterial smooth muscle cells. Am J Physiol Lung Cell Mol Physiol. 2013;305(4):L312‐L321. doi:10.1152/ajplung.00054.2013 23771884 PMC3891012

[46] Inman GJ , Nicolás FJ , Callahan JF , et al. SB‐431542 is a potent and specific inhibitor of transforming growth factor‐beta superfamily type I activin receptor‐like kinase (ALK) receptors ALK4, ALK5, and ALK7. Mol Pharmacol. 2002;62(1):65‐74. doi:10.1124/mol.62.1.65 12065756

[47] Williams L , Layton T , Yang N , Feldmann M , Nanchahal J . Collagen VI as a driver and disease biomarker in human fibrosis. FEBS J. 2022;289(13):3603‐3629. doi:10.1111/febs.16039 34109754

[48] Ma Y , Hossen MM , Huang JJ , et al. Growth arrest and DNA damage‐inducible 45: a new player on inflammatory diseases. Front Immunol. 2025;16:1513069. doi:10.3389/fimmu.2025.1513069 40083548 PMC11903704

[49] Murata H , Tanaka S , Okada H . The regulators of human endometrial stromal cell decidualization. Biomolecules. 2022;12(9):1275. doi:10.3390/biom12091275 36139114 PMC9496326

[50] Yang M , Ong J , Meng F , et al. Spatiotemporal insight into early pregnancy governed by immune‐featured stromal cells. Cell. 2023;186(20):4271‐4288.e24. doi:10.1016/j.cell.2023.08.020 37699390

[51] Yang J , Gong L , Liu Q , et al. Single‐cell RNA‐seq reveals developmental deficiencies in both the placentation and the decidualization in women with late‐onset preeclampsia. Front Immunol. 2023;14:1142273. doi:10.3389/fimmu.2023.1142273 37283740 PMC10239844

[52] Du L , Deng W , Zeng S , et al. Single‐cell transcriptome analysis reveals defective decidua stromal niche attributes to recurrent spontaneous abortion. Cell Prolif. 2021;54(11):e13125. doi:10.1111/cpr.13125 34546587 PMC8560595

[53] Brar AK , Frank GR , Richards RG , et al. Laminin decreases PRL and IGFBP‐1 expression during in vitro decidualization of human endometrial stromal cells. J Cell Physiol. 1995;163(1):30‐37. doi:10.1002/jcp.1041630105 7534770

[54] Zhang XH , Liang X , Liang XH , et al. The mesenchymal–epithelial transition during in vitro decidualization. Reprod Sci. 2013;20(4):354‐360. doi:10.1177/1933719112472738 23302397 PMC4077516

[55] Owusu‐Akyaw A , Krishnamoorthy K , Goldsmith LT , Morelli SS . The role of mesenchymal‐epithelial transition in endometrial function. Hum Reprod Update. 2019;25(1):114‐133. doi:10.1093/humupd/dmy035 30407544

[56] Purcell SH , Cantlon JD , Wright CD , Henkes LE , Seidel GEJr , Anthony RV . The involvement of proline‐rich 15 in early conceptus development in sheep. Biol Reprod. 2009;81(6):1112‐1121. doi:10.1095/biolreprod.109.076190 19605793 PMC2802235

[57] Gates KC , Cantlon JD , Goetzmann LN , Anthony RV . Proline rich 15 regulates trophoblast proliferation and differentiation. Biology of Reproduction. 2012;87(Suppl_1):378. doi:10.1093/biolreprod/87.s1.378

[58] Gates KC , Goetzmann LN , Cantlon JD , Jeckel KM , Anthony RV . Effect of proline rich 15‐deficiency on trophoblast viability and survival. PLoS One. 2017;12(4):e0174976. doi:10.1371/journal.pone.0174976 28380025 PMC5381842

[59] Xu J , Lamouille S , Derynck R . TGF‐beta‐induced epithelial to mesenchymal transition. Cell Res. 2009;19(2):156‐172. doi:10.1038/cr.2009.5 19153598 PMC4720263

[60] DeDiego ML , Martinez‐Sobrido L , Topham DJ . Novel functions of IFI44L as a feedback regulator of host antiviral responses. J Virol. 2019;93(21):e01159‐19. doi:10.1128/JVI.01159-19 31434731 PMC6803278

[61] Liu Y , Gao S , Zhao Y , Wang H , Pan Q , Shao Q . Decidual natural killer cells: a good nanny at the maternal‐fetal interface during early pregnancy. Front Immunol. 2021;12:663660. doi:10.3389/fimmu.2021.663660 34054831 PMC8149889

[62] Kumagai J , Kiuchi M , Kokubo K , et al. The USP7‐STAT3‐granzyme‐Par‐1 axis regulates allergic inflammation by promoting differentiation of IL‐5‐producing Th2 cells. Proc Natl Acad Sci U S A. 2023;120(49):e2302903120. doi:10.1073/pnas.2302903120 38015852 PMC10710068

[63] Stinson MW , Liu S , Laurenson AJ , Rotty JD . Macrophage migration is differentially regulated by fibronectin and laminin through altered adhesion and myosin II localization. Mol Biol Cell. 2024;35(2):ar22. doi:10.1091/mbc.E23-04-0137 38088893 PMC10881148

[64] Itoh M , Tamura A , Kanai S , et al. Lysosomal cholesterol overload in macrophages promotes liver fibrosis in a mouse model of NASH. J Exp Med. 2023;220(11):e20220681. doi:10.1084/jem.20220681 37725372 PMC10506914

[65] Large MJ , Wetendorf M , Lanz RB , et al. The epidermal growth factor receptor critically regulates endometrial function during early pregnancy. PLoS Genet. 2014;10(6):e1004451. doi:10.1371/journal.pgen.1004451 24945252 PMC4063709

[66] Peters VA , Joesting JJ , Freund GG . IL‐1 receptor 2 (IL‐1R2) and its role in immune regulation. Brain Behav Immun. 2013;32:1‐8. doi:10.1016/j.bbi.2012.11.006 23195532 PMC3610842

[67] McQuitty CE , Williams R , Chokshi S , Urbani L . Immunomodulatory role of the extracellular matrix within the liver disease microenvironment. Front Immunol. 2020;11:574276. doi:10.3389/fimmu.2020.574276 33262757 PMC7686550

[68] Jiang L , Fei H , Jin X , et al. Extracellular vesicle‐mediated secretion of HLA‐E by trophoblasts maintains pregnancy by regulating the metabolism of decidual NK cells. Int J Biol Sci. 2021;17(15):4377‐4395. doi:10.7150/ijbs.63390 34803505 PMC8579460

[69] Sonderegger S , Pollheimer J , Knöfler M . Wnt signalling in implantation, decidualisation and placental differentiation–review. Placenta. 2010;31(10):839‐847. doi:10.1016/j.placenta.2010.07.011 20716463 PMC2963059

[70] Mannion AJ , Odell AF , Taylor A , Jones PF , Cook GP . Tumour cell CD99 regulates transendothelial migration via CDC42 and actin remodelling. J Cell Sci. 2021;134(15):jcs240135. doi:10.1242/jcs.240135 34374417 PMC8403985

[71] Tang Z , Xia Z , Wang X , Liu Y . The critical role of osteopontin (OPN) in fibrotic diseases. Cytokine Growth Factor Rev. 2023;74:86‐99. doi:10.1016/j.cytogfr.2023.08.007 37648616

[72] Geijtenbeek TB , Gringhuis SI . Signalling through C‐type lectin receptors: shaping immune responses. Nat Rev Immunol. 2009;9(7):465‐479. doi:10.1038/nri2569 19521399 PMC7097056

[73] Tao X , Tao R , Wang K , Wu L . Anti‐inflammatory mechanism of apolipoprotein A‐I. Front Immunol. 2024;15:1417270. doi:10.3389/fimmu.2024.1417270 39040119 PMC11260610

[74] Zeng W , Wang F , Cui Z , et al. Inhibition of ferroptosis counteracts the advanced maternal age‐induced oocyte deterioration. Cell Death Differ. 2025;32(6):1071‐1085. doi:10.1038/s41418-025-01456-0 39910323 PMC12162888

[75] Mikwar M , MacFarlane AJ , Marchetti F . Mechanisms of oocyte aneuploidy associated with advanced maternal age. Mutat Res Rev Mutat Res. 2020;785:108320. doi:10.1016/j.mrrev.2020.108320 32800274

[76] Miao Y , Cui Z , Gao Q , Rui R , Xiong B . Nicotinamide mononucleotide supplementation reverses the declining quality of maternally aged oocytes. Cell Rep. 2020;32(5):107987. doi:10.1016/j.celrep.2020.107987 32755581

[77] Woods L , Perez‐Garcia V , Kieckbusch J , et al. Decidualisation and placentation defects are a major cause of age‐related reproductive decline. Nat Commun. 2017;8(1):352. doi:10.1038/s41467-017-00308-x 28874785 PMC5585348

[78] Llorca T , Ruiz‐Magaña MJ , Abadía AC , Ruiz‐Ruiz C , Olivares EG . Decidual stromal cells: fibroblasts specialized in immunoregulation during pregnancy. Trends Immunol. 2025;46(2):138‐152. doi:10.1016/j.it.2024.12.007 39947975

[79] Lee AWT , Ng JKW , Liao J , et al. Single‐cell RNA sequencing identifies molecular targets associated with poor in vitro maturation performance of oocytes collected from ovarian stimulation. Hum Reprod. 2021;36(7):1907‐1921. doi:10.1093/humrep/deab100 34052851

[80] Llonch S , Barragán M , Nieto P , et al. Single human oocyte transcriptome analysis reveals distinct maturation stage‐dependent pathways impacted by age. Aging Cell. 2021;20(5):e13360. doi:10.1111/acel.13360 33908703 PMC8135014

[81] Ntostis P , Iles D , Kokkali G , et al. The impact of maternal age on gene expression during the GV to MII transition in euploid human oocytes. Hum Reprod. 2022;37(1):80‐92. doi:10.1093/humrep/deab226 PMC8730309 34755188

[82] Timóteo‐Ferreira F , Abreu D , Mendes S , et al. Redox imbalance in age‐related ovarian dysfunction and perspectives for its prevention. Ageing Res Rev. 2021;68:101345. doi:10.1016/j.arr.2021.101345 33894395

[83] Vissers G , Giacomozzi M , Verdurmen W , Peek R , Nap A . The role of fibrosis in endometriosis: a systematic review. Hum Reprod Update. 2024;30(6):706‐750. doi:10.1093/humupd/dmae023 39067455 PMC11532625

[84] Zhang L , Tian J , Li N , et al. Exosomal miRNA reprogramming in pyroptotic macrophage drives silica‐induced fibroblast‐to‐myofibroblast transition and pulmonary fibrosis. J Hazard Mater. 2025;483:136629. doi:10.1016/j.jhazmat.2024.136629 39603130

[85] Yan X , Liu Z , Chen Y . Regulation of TGF‐beta signaling by Smad7. Acta Biochim Biophys Sin (Shanghai). 2009;41(4):263‐272. doi:10.1093/abbs/gmp018 19352540 PMC7110000

[86] Wu Y , Li M , Zhang J , Wang S . Unveiling uterine aging: much more to learn. Ageing Res Rev. 86:101879. doi:10.1016/j.arr.2023.101879 36764360

[87] Winkler I , Tolkachov A , Lammers F , et al. The cycling and aging mouse female reproductive tract at single‐cell resolution. Cell. 2024;187(4):981‐998.e25. doi:10.1016/j.cell.2024.01.021 38325365

[88] Fu B , Wei H . Decidual natural killer cells and the immune microenvironment at the maternal‐fetal interface. Sci China Life Sci. 2016;59(12):1224‐1231. doi:10.1007/s11427-016-0337-1 27905000

[89] Salminen A . Feed‐forward regulation between cellular senescence and immunosuppression promotes the aging process and age‐related diseases. Age Res Rev. 2021;67:101280. doi:10.1016/j.arr.2021.101280 33581314

[90] Tanner AR , Kennedy VC , Lynch CS , et al. In vivo investigation of ruminant placenta function and physiology‐a review. J Anim Sci. 2022;100(6):skac045. doi:10.1093/jas/skac045 35648127 PMC9159061

[91] Punzon‐Jimenez P , Machado‐Lopez A , Perez‐Moraga R , et al. Effect of aging on the human myometrium at single‐cell resolution. Nat Commun. 2024;15(1):945. doi:10.1038/s41467-024-45143-z 38296945 PMC10830479

