# Supplementary material for: Single‐cell transcriptome analyses reveal disturbed decidual microenvironment in women of advanced maternal age
Source: Clin Transl Med. 2025 Dec 17;15(12):e70541. doi: 10.1002/ctm2.70541 (PMC12711380; doi:10.1002/ctm2.70541)
Supplement: Supplementary file 1 — Supporting Information [file CTM2-15-e70541-s011.docx]

**SUPPLEMENTARY TABLES**

**TABLE S1. Clinical characteristics of pregnancies included in scRNA-seq analysis.**

| **Patient ID** | **Age** | **Gestational Sac**  **(cm^3^)** | **Gravidities** | **Miscarriges** | **Gestation (weeks)** | **BMI (kg/m^2^)** | **Group** |
| --- | --- | --- | --- | --- | --- | --- | --- |
| C01 | 22 | 2.16x1.14x1.92 | 1 | 1 | 6 | 19.38 | CTR |
| C02 | 24 | 2.01x0.76x2.01 | 0 | 0 | 6 | 19.25 | CTR |
| C03 | 26 | 2.59x1.68x1.13 | 0 | 1 | 6 | 20.44 | CTR |
| A01 | 35 | 2.27x1.74x2.03 | 2 | 3 | 6 | 20.7 | AMA |
| A02 | 36 | 1.75x0.94x2.21 | 2 | 0 | 6 | 19.57 | AMA |
| A03 | 36 | 2.53x1.35x2.43 | 2 | 3 | 6 | 20.57 | AMA |

CTR: Control; AMA: Advanced maternal age. Patient ID: Patient Identifier; BMI: Body Mass Index

**TABLE S2. Clinical characteristics of pregnancies included in validation analysis.**

| **Patient ID** | **Age** | **Gestational Sac**  **(cm^3^)** | **Gravidities** | **Miscarriges** | **Gestation (weeks)** | **BMI (kg/m^2^)** | **Group** |
| --- | --- | --- | --- | --- | --- | --- | --- |
| C04 | 30 | 1.24x0.68x1.42 | 2 | 0 | 7 | 19.38 | CTR |
| C05 | 28 | 3.13x0.94x1.25 | 0 | 0 | 6 | 20.44 | CTR |
| C06 | 28 | 2.33x1.37x1.81 | 2 | 1 | 6 | 23.19 | CTR |
| C07 | 29 | 1.55x0.34x1.10 | 2 | 2 | 6 | 19.53 | CTR |
| C08 | 22 | 1.4x0.92x1.03 | 0 | 0 | 6 | 21.33 | CTR |
| C09 | 22 | 2.53x0.7x1.7 | 0 | 0 | 6 | 19.39 | CTR |
| A04 | 35 | 1.42x1.02x1.46 | 1 | 1 | 6 | 20.44 | AMA |
| A05 | 38 | 1.62x0.98x2.12 | 4 | 1 | 7 | 20.57 | AMA |
| A06 | 36 | 3.10x2.25x1.63 | 1 | 1 | 9 | 20.71 | AMA |
| A07 | 36 | 1.67x1.14x1.10 | 4 | 2 | 7 | 21.23 | AMA |
| A08 | 35 | 2.58x0.98x2.86 | 3 | 1 | 8 | 20.94 | AMA |
| A09 | 37 | 1.94x1.41x1.03 | 3 | 1 | 6 | 19.67 | AMA |

**TABLE S3. Expansion medium composition for EEOs culture.**

| **Product** | **Company** | **Product Number** | **Final Concentration** |
| --- | --- | --- | --- |
| Advanced DMEM/F12 | Gibco | 12634028 | 1X |
| B27 supplement minus vitamin A | Life Technologies | 12587010 | 1X |
| Primocin | Invitrogen | ant-pm-1 | 100 μg/ml |
| N-Acetyl-L-cysteine | Sigma | A9165-5G | 1.25 mM |
| L-glutamine | Life Technologies | 25030-024 | 2 mM |
| Recombinant human EGF | SinoBiological | 10605-HNAE | 50 ng/ml |
| Recombinant human Noggin | SinoBiological | 10267-HNAH | 100 ng/ml |
| Recombinant human Rspondin-1 | SinoBiological | 11083-HNAS | 500 ng/ml |
| Recombinant human FGF-10 | SinoBiological | 10573-HNAE | 100 ng/ml |
| Recombinant human HGF | SinoBiological | 10463-HNAS | 50 ng/ml |
| ALK-4, -5, -7 inhibitor, A83-01 | MedChemExpress | HY-10432 | 500 nM |
| Nicotinamide | Sigma | N0636 | 10 nM |

**TABLE S4** Antibodies used in IHC, IF and WB.

| Antibody | Host species | Final concentration | Manufacturer | Catalogue number |
| --- | --- | --- | --- | --- |

| IGFBP1 | Mouse | WB 1:2000,  IHC 1;150 | OriGene Technologies | TA808733 |
| --- | --- | --- | --- | --- |
| p-SMAD3 | Rabbit | WB 1:1000 | Cell Signaling Technology | C25A9 |
| SMAD3 | Rabbit | WB 1:1000 | Cell Signaling Technology | C67H9 |
| p-SMAD2 | Rabbit | WB 1:1000 | Cell Signaling Technology | E8F3R |
| SMAD2 | Rabbit | WB 1:1000 | Cell Signaling Technology | D43B4 |
| COL6A1 | Mouse | WB 1:1000 | Abmart | T55912 |
| GAPDH | Mouse | WB 1:5000 | Beyotime | AF0006 |
| αTubulin | Rabbit | WB 1:1000 | Beyotime | AF0001 |
| Vimentin | Rabbit | WB 1:1000  IF 1:200 | Abcam | ab92547 |
| Cytokeratin 7 | Mouse | IF 1:200 | Proteintech | 66483-1-Ig |
| E-cadherin | Mouse | IF 1:200 | Abcam | ab76055 |
| Laminin | Rabbit | IF 1:100 | Abcam | ab133645 |
| EPCAM | Mouse | IF 1:200 | Proteintech | 66316-1-Ig |
| CD14 | Mouse | IF 1:200 | Proteintech | 60253-1-Ig |
| CD56 | Rabbit | IF 1:200 | Proteintech | 14255-1-AP |
| αSMA | Rabbit | IF 1:200 | Proteintech | 14395-1-AP |
| COL1A1 | Rabbit | IHC 1:1500 | Abcam | ab138492 |
| SMAD2/3 | Rabbit | IF 1:100 | Cell Signaling Technology | 5678S |
| PDGFRA | Rabbit | IF 1:200 | OriGene | TA379744S |
| PRL | Mouse | IF 1:100 | OriGene | TA500719 |
| PRLR | Rabbit | IF 1:200 | OriGene | TA423249S |
| TGFB1 | Rabbit | IF 1:200 | OriGene | AP06350PU-N |
| TGFBR1 | Rabbit | IF 1:200 | OriGene | TA385400S |

**Table S5. Sequences used for PRR15 overexpression constructs**

| **Construct** | **Sequence (5’-3’)** |
| --- | --- |
| PRR15 Overexpression | 5′-ATGGCCGACAGCGGCGATGCTGGCAGCTCCGGCCCCTGGTGGAAATCGCTCACCAACAGCAGAAAGAAAAGCAAGGAAGCCGCAGTGGGGGTGCCGCCTCCCGCCCAGCCCGCTCCCGGGGAGCCCACGCCACCTGCGCCGCCCAGCCCGGACTGGACCAGCAGCTCCCGGGAGAACCAGCACCCCAATCTCCTCGGGGGCGCCGGCGAGCCCCCCAAACCAGACAAGTTATACGGGGACAAATCCGGCAGCAGCCGCCGCAATTTGAAGATCTCGCGCTCCGGCCGCTTTAAGGAGAAGAGGAAAGTGCGCGCCACGCTGCTCCCGGAGGCGGGCAGGTCCCCGGAGGAGGCAGGCTTTCCTGGTGACCCCCACGAGGACAAGCAGTAG-3′ |
| Vector Control | 5′-CTCAAGCTTCGAATTCCTAGGCCCG-3′ |
|  |  |

**TABLE S6. Primer sequences for RT-qPCR**

| **Primer Name** | **Sequence (5’-3’)** |
| --- | --- |
| *ACTB* | 5’ CATGTACGTTGCTATCCAGGC 3’  5’ CTCCTTAATGTCACGCACGAT 3’ |
| *IGFBP1* | 5’ GCCCAGAGAGCACGGAGATAAC 3’  5’ GGAGAGCCTTCGAGCCATCATAG 3’ |
| *PRL* | 5’ AGACAAGGAGCAAGCCCAACAG 3’  5’ CTTCCGTGACCAGATGATACAGAGG 3’ |
| *PRR15* | 5’ GGAGCCCACGCCACCTG 3’  5’ CCCCGTATAACTTGTCTGGTTTGG 3’ |
| *ACTA2* | 5’ CTATGCCTCTGGACGCACAACT 3’ |
|  | 5’ CAGATCCAGACGCATGATGGCA 3’ |
| *COL1A1* | 5’ GATTCCCTGGACCTAAAGGTGC 3’ |
|  | 5’ AGCCTCTCCATCTTTGCCAGCA 3’ |
|  |  |

**TABLE S7. The cell number and quality analysis of scRNA-seq data in each sample**

| **Samples** | **C01** | **C02** | **C03** | **A01** | **A02** | **A03** |
| --- | --- | --- | --- | --- | --- | --- |
| Group | CTR | CTR | CTR | AMA | AMA | AMA |
| Estimated Cells | 16,457 | 12,401 | 13,625 | 18,731 | 12,946 | 14,114 |
| Fraction Reads in Cell (%） | 83.8 | 91.4 | 87.6 | 85.6 | 85.9 | 86.6% |
| Mean Reads per Cell3 | 34,035 | 54,087 | 47,417 | 32,843 | 36,218 | 35,500 |
| Median Genes per Cell | 3,178 | 4,352 | 3,349 | 1,677 | 3,643 | 3,847 |
| Total Genes Detected | 31,369 | 31,782 | 31,278 | 31,861 | 31,270 | 30,812 |
