# Supplementary figures and images for: Single‐cell transcriptome analyses reveal disturbed decidual microenvironment in women of advanced maternal age
Source: Clin Transl Med. 2025 Dec 17;15(12):e70541. doi: 10.1002/ctm2.70541 (PMC12711380; doi:10.1002/ctm2.70541)

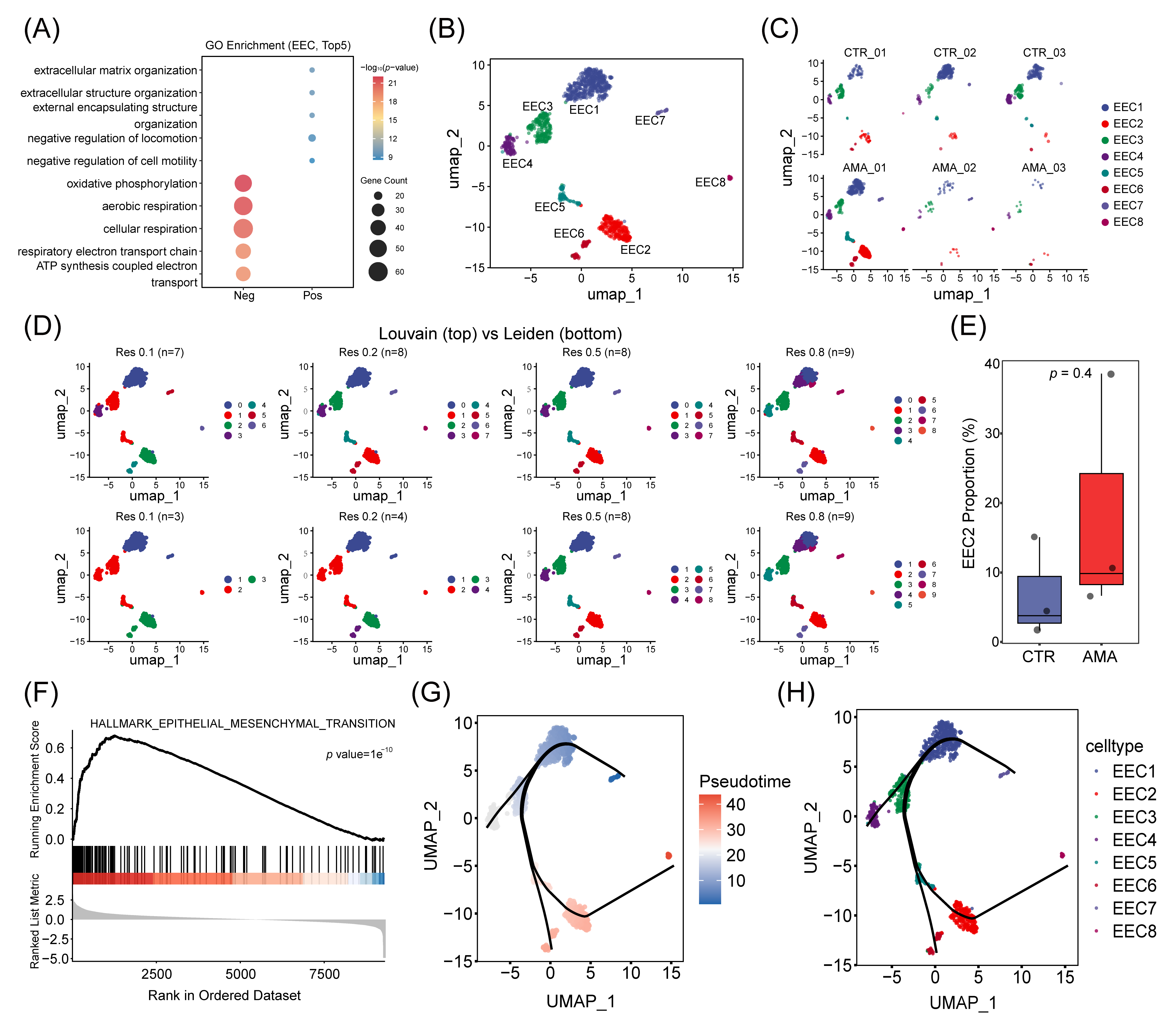

Supplement: Supplementary file 11 — Supporting Information [file CTM2-15-e70541-s012.tif]

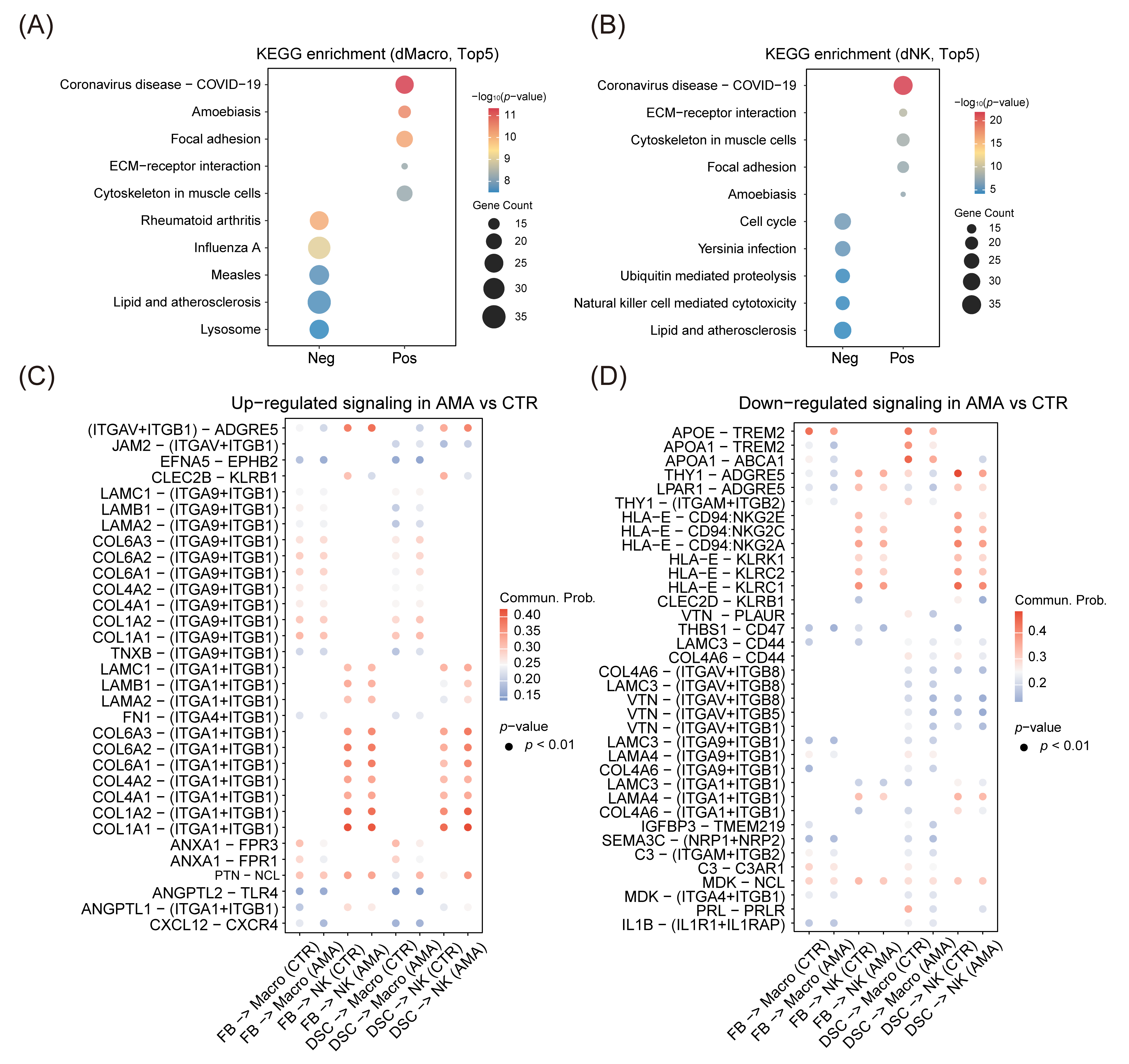

Supplement: Supplementary file 12 — Supporting Information [file CTM2-15-e70541-s010.tif]
