# Supplementary material for: Single‐cell transcriptome analyses reveal disturbed decidual microenvironment in women of advanced maternal age
Source: Clin Transl Med. 2025 Dec 17;15(12):e70541. doi: 10.1002/ctm2.70541 (PMC12711380; doi:10.1002/ctm2.70541)
Supplement: Supplementary file 14 — Supporting Information [file CTM2-15-e70541-s006.docx]

**FIGURE S1 Characterisation of decidual dysfunction in women of AMA.** (A) A magnified H&E-stained section of the decidual stromal compartment from the CTR and AMA group. The arrow indicates a region exhibiting features of defective stromal cell differentiation, including nuclear pleomorphism and architectural disorganisation. 100 μm (left panel) and 50 μm (right panel). (B) PRL mRNA levels in decidualised EnSCs measured by RT-qPCR (n=6 biological replicates per group). (C) Immunofluorescence staining of decidua and organoid for E-cadherin (E-cad), Cytokeratin-7 (CK7), epithelial cell adhesion molecule (EPCAM), and basement membrane marker Laminin (LAM). Scale bars: 50 μm (decidua); 250 μm (organoids). (D) Representative bright-field images of CTR- and AMA-derived EEOs on day 7 of passage 0 after seeding. Scale bar: 500 μm. (E) Quantification of the EEO growth rate, calculated from the change in area between day 5 and day 6 using the formula: growth rate = (area day6 – area day5) / area day5) × 100% (n=3 biological replicates per group, with 4 organoids measured per patient). Data in bar graphs are presented as the mean ± SD. Statistical significance was determined by a two-tailed unpaired Student's t-test (***p < 0.01*, ****p < 0.001*).

**FIGURE S2 Quality control and cellular composition analyses of decidual samples.** (A) Distribution of unique molecular identifier (UMI) counts per cell, gene counts per cell and the percentage of mitochondrial transcripts per cell detected in the CTR and AMA decidual samples. (B) UMAP visualization of all 62,758 cells, identifying 12 distinct cell populations. (C) UMAP visualization split by individual sample, showing consistent cluster positions and minimal batch effects. (D) Dot plot showing expression of canonical marker genes. (E) KEGG enrichment analysis (Top 5 pathways) for upregulated (Pos) and downregulated (Neg) genes. (F) Bar plot showing the relative cell type proportions for each of the 6 individual patient samples from the discovery cohort. (G) Additional representative IHC images for COL1A1 in the validation cohort. Scale bar: 50 μm. (H) Representative IF staining for macrophages (marked by CD14, red) in the validation cohort. Scale bar: 50 μm. (I) Quantification of CD14⁺ cell proportion from IF images (n=6 biological replicates per group). (J) Representative IF staining for NK cells (marked by CD56, red) in the validation cohort. Scale bar: 50 μm. (K) Quantification of CD56⁺ cell proportion from IF images (n=6 biological replicates per group). Data are presented as the mean ± SD. Statistical significance was determined by a two-tailed unpaired Student's t-test (***p < 0.01,* ****p* < 0.01).

**FIGURE S3 Fibroblast subclustering validation and functional analysis.**  (A) GO and KEGG enrichment analysis (Top 5 pathways) of upregulated DEGs in the global FB population. (B) Integrated UMAP visualization of all fibroblast subclusters. (C) UMAP visualization of fibroblast subclusters colored by individual patient samples. (D) Computational validation of the FB3 cluster using alternative clustering resolutions and the Leiden algorithm. (E) The relative proportions of fibroblast subclusters between CTR and AMA groups (n=3 per group, *p* > 0.05). (F) KEGG pathway enrichment (Top 5 pathways) of upregulated and downregulated DEGs within each FB subcluster. (G) Dot plot showing the average expression (color scale) and percentage of expressing cells (dot size) for key TGF-β pathway negative regulators. The analysis compares CTR and AMA conditions across the three identified fibroblast subsets (FB1, FB2, and FB3). (H, I) Global trajectory inference visualized on the integrated UMAP embedding, with cells colored by pseudotime (H) and cell type (I). (J, K) RT-qPCR analysis of *COL1A1* (J) and *ACTA2* (K) mRNA levels in the expanded decidual cohort (n=6 biological replicates per group). (L, M) RT-qPCR analysis of fibrotic gene expression in primary fibroblasts from AMA donors. The bar graphs show the relative mRNA expression of (L) *COL1A1* and (M) *ACTA2* following treatment with the TGF-β receptor I inhibitor, SB431542 (10 µM) (n=6 biological replicates per group). Data are presented as the mean ± SD. Statistical significance was determined by a two-tailed unpaired Student's t-test (**p* < 0.05, ***p* < 0.01, ****p*< 0.001, **** *p*< 0.0001).

**FIGURE S4 Characterization and heterogeneity of decidual stromal cell subsets**. (A) Violin plots displaying the normalized expression of key upregulated (pro-fibrotic/stress) and downregulated (decidualisation) genes in the global DSC population from AMA versus CTR groups. (B) UMAP plots of DSC subsets split by individual patient samples, demonstrating consistent clustering across biological replicates. (C) Integrated UMAP visualization of all DSC subsets. (D) Assessment of DSC clustering stability using a range of resolution parameters (left) and the alternative Leiden clustering algorithm (right). (E) Dot plot displaying the expression of selected marker genes defining each of the four DSC subsets. (F) Bar plot showing the relative proportions of the four DSC subsets in CTR and AMA groups (*p* > 0.05, two-sided Wilcoxon rank-sum test). (G, H) Global trajectory inference of DSC differentiation visualized on the integrated UMAP, with cells colored by their inferred pseudotime value (G) and subtype (H). (I, J) Smoothed line plots visualizing the expression dynamics of key gene clusters (decidualisation markers *IGFBP1*, *PRL*, *PRR15* (I) and pro-fibrotic/stress markers *GADD45A*, *ATF3*, *IGFBP5* (J) along the Lineage 1 pseudotime axis, compared between CTR and AMA groups. (K, L) Binned expression analysis (box plots) confirming that, along Lineage 1, AMA cells show lower expression of decidualisation markers (*IGFBP1*, *PRL*, *PRR15*) (K) and significantly higher expression of pro-fibrotic/stress markers (*GADD45A*, *ATF3*, *IGFBP5*) (L). (M, N) Spearman correlation plots comparing the mean *PRR15* expression (X-axis) against the meanfunctional scores (Y-axis) for decidualisation (M) and collagen production (N). Scores were derived from the pan-DSC population for each biological replicate. Each point represents one patient sample (n=3 CTR, blue circles; n=3 AMA, red triangles). Spearman rho and *p*-values are indicated on each plot. Violin plots (A), box plots (K, L) and spearman correlation plots (M, N) statistical significance was determined by a Wilcoxon rank-sum test (**** *p* < 0.0001).

**FIGURE S5 Supporting data for PRR15 functional analysis.** (A) Representative bright-field (left) and fluorescence (right) images of stromal cells transduced with KD-NC or KD-PRR15 lentivirus. Cells were subsequently treated with MPA+cAMP to induce decidualisation. The bright-field images show that KD-PRR15 cells retained a fibroblastic morphology, indicating decidualisation failure. ZsGreen fluorescence confirms successful lentiviral transduction in both groups. Scale bar, 200 μm. (B) RT-qPCR analysis of *IGFBP1* mRNA expression at indicated time points in KD-NC- and KD-PRR15-transfected cells undergoing in vitro decidualisation, (n=4 biological replicates). (C, D) Western blot (C) and quantification (D) analysis of SMAD2 and p-SMAD2 expression in stable knockdown cell lines (n=4 biological replicates). (E) ELISA (rescue experiment) analysis of secreted IGFBP1 protein in the medium from KD-NC and KD-PRR15 cells treated with SB431542 (10 µM) (n=3 biological replicates). (F, G) Representative IF images (F) and quantification (G) of the proportion of nuclear SMAD2/3 (green) positive cells, showing SB431542 blocks knockdown-induced nuclear translocation (n=3 biological replicates). Scale bar: 50 μm. (H) RT-qPCR analysis of *PRR15* overexpression (OE-PRR15) efficiency (n=3 biological replicates). (I) RT-qPCR analysis showing *PRR15* overexpression (OE-PRR15) enhances *IGFBP1* mRNA expression (n=3 biological replicates). (J) ELISA of secreted TGF-β1 protein in the culture medium from cells treated as in (H) (n=3 biological replicates). (K, L) RT-qPCR analysis showing *PRR15* overexpression leads to the downregulation of key fibrotic target genes, *ACTA2* (K) and *COL1A1* (L) (n=3 biological replicates). Data are shown as the mean ± SD and were analysed by a two-tailed unpaired Student's t-test or one-way ANOVA. (**p* < 0.01, ***p* < 0.001, ****p* < 0.0001; ns, not significant).

**FIGURE S6 Characterization of EEC subsets and EMT dynamics. (A) Enriched GO terms (Top 5 pathways) for genes upregulated (Pos) and downregulated (Neg) in the global EEC population of AMA samples. (B, C) UMAP plots of the eight EEC subsets, shown for the integrated dataset (B) and split by individual patient samples (C). (D) Assessment of clustering stability. The annotation into eight EEC subsets remained consistent across a range of resolution parameters (left) and when using the alternative Leiden clustering algorithm (right). (E) Bar plot showing the relative proportions of the EEC2 subset in CTR and AMA groups (n=3 per group; p = 0.4, Wilcoxon rank-sum test). (F) Gene Set Enrichment Analysis (GSEA) plot showing significant enrichment (p-value = 10^-10^) of the 'HALLMARK_EPITHELIAL_MESENCHYMAL_TRANSITION' pathway in the EEC2 subset (AMA vs. CTR). (G, H) Global trajectory inference of EEC differentiation visualized on the integrated UMAP by Slingshot, with cells colored by their inferred pseudotime value (G) and subtype (H).**

**FIGURE S7 | Functional enrichment analysis of decidual immune cells. (**A, B) **Enriched KEGG terms (Top 5 pathways) for genes upregulated (Pos) and downregulated (Neg) in the global dMacro (A) and dNK (B) population from women of AMA compared with CTR group.** (C) Dot plot showing significantly upregulated signalling pathways (*p* < 0.01) originating from stromal cells (FB, DSC) and targeting immune cells (Macro, NK) in AMA. (D) Dot plot showing significantly downregulated signalling pathways (*p* < 0.01) originating from stromal cells and targeting immune cells in AMA.

**FIGURE S8** **Dysregulated cell-to-cell communication in the AMA group.** (A) Bar plots showing a global reduction in the total number of inferred interactions and overall interaction strength in the AMA group compared to CTR. (B) Heatmaps showing the differential number of interactions and interaction strength by source (sender) cell type. (C, D) Chord plots (C) and relative contribution analysis (D) of the EGF signalling pathway. (E, F) Chord plots (E) and relative contribution analysis (F) of the WNT signalling pathway. (G, H) Chord plots (G) and relative contribution analysis (H) of the PTN signalling pathway. (I, J) Chord plots (I) and relative contribution analysis (J) of the MDK signalling pathway. (K, L) Chord plots (K) and relative contribution analysis (L) of the CLEC signalling pathway. (M, N) Chord plots (M) and relative contribution analysis (N) of the ApoA signalling pathway.
